# When it comes to assessing the impact of e-cigarettes, estimates of device prevalence matter: the BIDI Stick disposable device

**DOI:** 10.1186/s12954-023-00820-y

**Published:** 2023-07-05

**Authors:** Neil McKeganey, Andrea Patton, Venus Marza, Gabriel Barnard

**Affiliations:** Centre for Substance Use Research, 4 Woodside Terrace, Glasgow, G3 7UY UK

**Keywords:** E-cigarettes, Youth young adult use, Prevalence, BIDI Stick

## Abstract

**Background:**

While e-cigarettes have been identified as an effective means of tobacco harm reduction, the degree to which these devices will realise their harm reduction potential will be determined in large part by how available these products are to adults who smoke. One of the key factors determining that availability is the regulatory regime around these products. Within the US e-cigarettes have become the most commonly used tobacco product by middle and high school students, with disposable e-cigarettes now the most popular type of device used by youth. In this paper, we report data on the prevalence with which one of the most popular disposable e-cigarettes within the US is being used by youth (aged 13–17) and underage young adults (aged 18–20).

**Methods:**

A cross-sectional online survey of a probability-based sample of 1215 youth (13–17 years) recruited via Ipsos’ KnowledgePanel and 3370 young adults aged 18–24, among whom 1125 were aged 18–20, recruited via Ipsos’ KnowledgePanel and online consumer research panels.

**Results:**

Among youth, 3.50% (95% CI: 2.46–4.83) reported smoking combustible cigarettes in the past 30 days, and 6.73% (95% CI: 5.26–8.48) reported using an e-cigarette in the past 30 days. Among underage young adults, 7.22% (95% CI: 5.24–9.67) reported smoking combustible cigarettes every day or some days, and 15.90% (95% CI: 12.96–19.21) reported using e-cigarettes every day or some days. Despite the scale of e-cigarette use in general among the US youth, only 0.04% (95% CI: 0.00–0.38) of 13–17 years old reported using the BIDI® Stick disposable e-cigarette in the past 30 days.

**Conclusions:**

While disposable e-cigarettes have become the most popular type of e-cigarette used by the US youth, it is likely that the prevalence of use of individual devices varies significantly. There is a need to regularly monitor the use of e-cigarettes by type of device and brand, in order to determine which products have the greatest potential to reduce smoking-related harm among adults who smoke and which products are being used by youth and underage young adults.

**Supplementary Information:**

The online version contains supplementary material available at 10.1186/s12954-023-00820-y.

## Introduction

In 2022, the global market for e-cigarettes was estimated to be worth $22.8 billion, projected to rise at an annual rate of 4.3% from 2022 to 2027 [1]. Although there are no precise figures for the number of people using e-cigarettes, that figure has been estimated to be in the region of 81.9 m in 2021 [2]. As the number of people using e-cigarettes has increased, so too has the commitment on the part of national and international regulatory bodies in determining whether these devices are having a positive or negative impact on public health, and how they ought to be regulated [3]. In a review undertaken by the internationally respected Cochrane Centre, the capacity of e-cigarettes to assist adults who smoke in quitting was clearly recognized:

People are more likely to stop smoking for at least 6 months using nicotine e-cigarettes than using nicotine replacement therapy or e-cigarettes without nicotine. They may work better than no support or behavioral support alone, and they may not be associated with serious unwanted effects [4].

An earlier review of the evidence on the health impact of e-cigarettes undertaken by the US National Academies of Science Engineering and Medicine similarly concluded that:

There is substantial evidence that completely switching from regular use of combustible tobacco cigarettes to e-cigarettes results in reduced short-term adverse health outcomes in several organ systems [5].

Recent research has shown that where adults who smoke have access to e-cigarettes, their use is associated with an increased number of quit attempts and an increased likelihood that those quit attempts will have been successful [6–9]. While the long-term harms associated with e-cigarettes remain unknown at present, and are likely to remain so for many years to come, these devices have been judged to be substantially less harmful than combustible tobacco products by a range of respected public health bodies, including: the UK Office for Health Improvement and Disparities, the UK Royal College of Physicians, and the Royal College of General Practitioners, and Cancer Research UK [10–12].

On the basis of what is now a substantial body of evidence, e-cigarettes have the potential to reduce smoking-related harm at the population level and at the level of the individual who is smoking. Importantly, that capacity exists wherever smoking combustible tobacco products occur, irrespective of the population group involved, and independently of the cultural context within which that smoking is occurring. However, whether these devices will achieve that potential relies to a large extent on the degree to which they are available for use by those who are smoking, and the extent to which those who are smoking choose to use these devices as an alternative to combustible cigarettes. The fact that the estimated total number of people using e-cigarettes is less than 10% of the estimated total number of people smoking globally (1.1B) is an illustration of how far e-cigarettes are from realizing that potential [13].

There are multiple reasons why these devices remain unavailable to millions of adults who smoke globally, and there are many reasons why, even in those areas where these devices are widely available, a large proportion of adults who smoke choose not to use these devices. One factor explaining why some people may choose not to use these devices is the erroneous perception that e-cigarettes are as harmful, if not more harmful, than combustible tobacco products. Research has shown that where such misperceptions occur, there is a reduced likelihood that these products will be used by those who are smoking [14, 15].

In relation to the degree to which e-cigarettes are available for use by those who are smoking, one of the major determinants is the question of how these devices are regulated by national and international bodies. In some countries (e.g., Brazil, Singapore, and India), access to these devices is entirely prohibited, while in other countries, access is limited to those in receipt of a medical prescription (e.g., Australia). In striking contrast to such restrictive regulatory action, some countries (e.g., Switzerland and the UK) have opted to ensure that adults who are smoking have relatively easy access to these products. Within the UK, so convinced is the government of the positive impact these devices in assisting adults who smoke in quitting that a scheme was recently announced enabling adults who are smoking to exchange their combustible cigarettes for e-cigarettes free of charge [16].

If there is one thing that has shaped the regulatory environment around these products more than any other, it is the use of these devices by young people. Within the US, the growth in the numbers of young people using e-cigarettes has been described by public health leaders as an “epidemic.” [17]. Although the number of the US youth reporting having used an e-cigarette in the past 30 days has recently reduced, nevertheless concern remains high at the overall level of e-cigarette use among youth and the marked recent growth in popularity of disposable e-cigarettes [18, 19]:

In 2022, 14.1% of high school students and 3.3% of middle school students reported past 30-day (current) e-cigarette use. Among those who used e-cigarettes in the past 30 days, the types of devices most often used were disposables (high school = 57.2% and middle school = 45.8%), followed by prefilled or refillable pods or cartridges (high school = 25.7% and middle school = 21.6%), and tanks or mod systems (high school = 5.9% and middle school = 9.8%), with 11.2% of high school students and nearly 23% of middle school students reporting not knowing the type of e-cigarette device used [20].

According to the 2022 National Youth Tobacco Survey (NYTS), the Puff Bar disposable flavored e-cigarette has become the most popular device used by middle and high school pupils within the US. Among those pupils who report having used an e-cigarette in the past 30 days, 29.7% identified the Puff Bar device as the e-cigarette they had used [20]. In advance of the 2022 NYTS, and following publication of the survey findings, there has been mounting concern at the increasing appeal and use of disposable e-cigarettes by the US youth to the point that these products have become virtually synonymous with youth vaping and public health harm [21–24]. Within much of the critical commentary around disposable e-cigarettes, there is an assumption: a) that all disposable e-cigarettes pose the same level of harm in terms of youth use, and b) that these products offer no notable public health benefit in terms of their capacity to assist adults who smoke in quitting. Within a context of mounting concern, the US Food and Drug Administration (FDA) has issued warning letters to the manufacturers of disposable e-cigarette products:

Today, the US Food and Drug Administration issued warning letters notifying ten companies, including Cool Clouds Distribution Inc. (doing business as Puff Bar), to remove their flavored disposable e-cigarettes and youth-appealing e-liquid products from the market because they do not have the required premarket authorization. These new actions are part of the FDAs ongoing, aggressive effort to act against illegally marketed tobacco products amid the public health crisis of youth e-cigarette use in America. The agency is particularly concerned about the appeal of flavored, disposable e-cigarettes to youth and continues to monitor all available data [25].

In addition to such warning letters, the FDA has issued “Marketing Denial Orders” to a range of disposable e-cigarette manufacturers on the basis that these products are judged to not be “appropriate for the protection of the public health” [26]. Disposable e-cigarettes, it could be said, have become the “new JUUL,” characterized by influential political figures, public health leaders, the media, and local lobby groups as the driver of public health harm and youth vaping [27–29].

Within a context of such heightened fears, it has become increasingly important to establish the extent to which individual, named, e-cigarette devices including disposable devices and e-liquids, are  being used by youth and underage young adults within the US. While the NYTS has reported on the extent to which the Puff Bar device is being used by the US youth, other brands of disposable e-cigarette devices are included within this survey only as a “write-in” category, requesting information on “some other brands not listed here.” As a result, there is a serious lack of information as to how widely specific brands of e-cigarettes are being used by youth and underage young adults within the US—information which is essential to establishing the public health impact of these devices.

In the remainder of this paper, we report on research designed to estimate the prevalence with which the second most popular disposable e-cigarette within the US (BIDI® Stick) is being used by youth and underage young adults. According to recent estimates, the BIDI® Stick range of flavored disposable e-cigarettes occupies 24.2% of the disposable e-cigarette market within the US [30]. The manufacturer of this product received a Marketing Denial Order in 2021 which, in 2022, was set aside following a decision by the US Court of Appeals for the Eleventh Circuit, to request the FDA to reassess the evidence which the original Marketing Denial Order was based upon [31].

## Methods

This study was an online, cross-sectional, self-report survey administered to national probability-based samples of 1215 youth aged 13–17 years and 3,370 young adults aged 18–24 years in the United States (US) in June 2022. Probability-based sampling increases the representativeness of the sample because each respondent has an equal chance of being included within the sample. As a result, the prevalence of use estimates based upon probability-based sampling can be generalized to the wider population of youth and young adults in the US.

Youth survey respondents were all children of adult panel members of the *Ipsos-Insight, LLC (“Ipsos”)* KnowledgePanel, which is the largest, probability-based, internet research panel in the US designed to be representative of the non-institutionalized US population aged 18 years and older. Young adults are a subpopulation that is under-represented in KnowledgePanel; therefore, a blended sample from both KnowledgePanel and non-probability online (“opt-in”) panels was used [32]. Young adult respondents were either members of Ipsos’ KnowledgePanel (25% of survey participants) or members of non-probability opt-in panels maintained by Ipsos and its sample partners. To align the probability and non-probability samples, Ipsos utilize a calibration methodology to correct for biases due to systematic under-coverage associated with non-probability samples from opt-in panels. As compared to samples that exclusively reply on non-probability samples without calibration, this blended sample represents the target population more effectively and offers more robust inferential possibilities. The representativeness is improved with respect to geodemographic distributions, as well as an important set of attitudinal/behavioral measures.

A total of 4256 invitations were sent out to adult members of KnowledgePanel who had a child aged 13–17 years living in their household of which 1215 (28.5%) completed the questionnaire. A total of 6810 invitations were sent out to young adult members of KnowledgePanel or online opt-in panels aged 18–24 years of which 3370 (49.5%) completed the questionnaire. Among the 3370 young adults participating in this survey, 1125 were aged 18–20, i.e., below the legal age (21) at which tobacco products can be purchased within the US. It is the sub-sample of the young adults, along with those youth aged 13–17 who are the focus of this paper.

In recruiting the youth sample, KnowledgePanel members who were parents were sent a screening questionnaire by Ipsos to verify that the individual panel member was a parent of a child aged 13–17 years old, that the child was living in the parent’s household, and that the parent was willing for their child to take part in the study. The parent was shown a consent form and when a parent provided consent for their child to take part, the parent was asked to have their child read the youth assent form. Young adult participants were provided with an informed consent form. Consent forms provided to parents, youth and young adults outlined the nature of the research being undertaken, the reason why the research was being undertaken, the type of information that would be requested, and the fact that survey participants would be shown images of tobacco products. It was further clarified to potential survey participants that their involvement in the research was entirely voluntary and that they would be free at any point to withdraw from the research.

Participants self-completed a web-based survey instrument composed predominately of questions and response options that were extracted or adapted from two established national, annually administered, surveys of tobacco use behaviors, perceptions, and intentions among middle and high school students in the US: (i) the 2021 National Youth Tobacco Survey (NYTS) and (ii) the Youth Interview Form administered at Wave 5 of the Population Assessment of Tobacco and Health (PATH) Study. NYTS is a national school-based survey that collects data on tobacco use by middle and high school students in the US and is a collaboration between the Centers for Disease Control and Prevention, Office on Smoking and Health (CDC, OSH) and the Food and Drug Administration, Center for Tobacco Products (FDA, CTP) [33]. The PATH study, a national longitudinal study of tobacco use in the US, is a collaboration between the National Institute for Health (NIH) and the FDA [34]. NYTS data are routinely cited by the Center for Tobacco Products when reporting the outcome of their determination of whether a tobacco product is “appropriate for the protection of the public health,” the legally required standard for deciding whether these products can be legally sold within the US as set out in the Family Smoking Prevention and Tobacco Control Act 2009 [35].

In assessing survey participants’ use and intentions to use cigarettes and e-cigarettes, the format and wording of questions, and response options, were extracted exactly or near-exactly as they appeared in these instruments with the only modification being the inclusion of individual product names. To increase the accuracy of the reporting with regard to which e-cigarette brands and devices respondents reported having used, narrative questioning based upon the NYTS and PATH instruments was combined with the presentation of device and brand images.

### Measures

#### Demographic information

Demographic information recorded in the survey encompassed age, gender, ethnicity, race, school grade/education level, household income, and residence state.

### Awareness and use of any type of e-cigarette

Before participants were shown questions relating to e-cigarette use, the following text was displayed on screen, “The next several questions are about electronic cigarettes or e-cigarettes, such as JUUL, Vuse, blu, and Logic. E-cigarettes are battery powered devices that usually contain a nicotine-based liquid that is vaporized and inhaled. You may also know them as e-cigs, vape-pens, e-hookahs, vapes, or mods.” Awareness of e-cigarettes was assessed by the question “Have you ever seen or heard of e-cigarettes before this study?” Participants who answered “No” were routed to the end of the survey. Participants who answered “Yes” were asked the question, “Have you ever used an e-cigarette, even one or two times?” Participants who answered “Yes” to this question were routed via logic to questions about the age at which they first used an e-cigarette, the number of times they have used an e-cigarette in their lifetime, the number of days in the past 30 days that they used an e-cigarette, and when they last used an e-cigarette. Young adults were also asked whether they now use an e-cigarette “Every day,” “Some days,” or “Not at all”.

### Use of e-cigarette brands

Those who reported having ever used an e-cigarette were routed via logic to questions about e-cigarette brands they had ever used. Survey participants were shown images of 19 different e-cigarette brand logos and asked “Please look carefully at the brand logos below. Have you ever used any of these brands of e-cigarettes, even once or twice? (Select all that you have ever used).” The 19 images of brand logos were shown across four questions. The order of the brand logos within each question was randomized as was the order of the four brand questions. In each question, participants could select “I have not used any of these brands of e-cigarettes”. Participants who had never used any of these 19 e-cigarette brands were routed to the end of the survey. Participants who had used one or more of the 19 e-cigarette brands were routed to a section about use of individual devices from the brands they reported to having used.

### Ever use of specific e-cigarette devices

Participants who reported having ever used an e-cigarette brand were asked the name of the device they had ever used in response to the question “You said that you have used a [brand name] e-cigarette. Below are the pictures of different e-cigarettes that are made by [brand name]. Which of these [brand name] e-cigarettes have you ever used, even once or twice (Check all that apply).” The order in which images of individual e-cigarette devices were presented to respondents was randomized, and participants could select “I have not used any of these e-cigarettes” in response to the images presented.

Participants who reported having ever used a device from that brand were asked the number of times they had used that device in their entire life using the question “How many times have you used the [brand and device name] in your entire life?” Participants could select from a list of six response options ranging from “1 time, even just a few puffs” to “100 or more times.” Young adult participants were also asked if they now used the device “Every day,” “Some days,” or “Not at all.” In both questions, participants were shown an image of the device.

### Past 30-day use of e-cigarette brands

Participants who reported that they had used e-cigarettes in the past 30 days and reported having ever used one or more of the 19 e-cigarette brands were asked about their use of these e-cigarette brands during the past 30 days using the question, “Please look carefully at the brand logos below. In the past 30 days, have you used any of these brands of e-cigarettes, even once or twice? (Select all that you have ever used).” Response options were dependent upon the selection(s) made by the participant previously in the survey when asked to select the e-cigarette brand(s) they had ever used. Only the brand logo(s) selected as part of the ever use question were available for selection in this question. The list of response options was randomized, and participants were given the option to respond, “I have not used any of these brands of e-cigarettes in the past 30 days.”

### Past 30-day use of e-cigarette devices

Youth participants who reported using an e-cigarette brand in the past 30 days and having ever used one of the devices from that brand were asked “Below are pictures of different [brand name] e-cigarettes that you said you have used. In the past 30 days, have you used any of these [brand name] e-cigarettes, even once or twice? (Check all that apply).” Response options were dependent upon the selection(s) made by the participant previously in the survey when asked to select the e-cigarette devices from that brand they had ever used. Only the devices selected as part of the ever use question were available for selection in this question. The response option images were randomized, and participants were given the option to respond, “I have not used any of these e-cigarettes in the past 30 days.” (Additional Files 1 and 2).

### Statistical analysis

Following data collection, design weights were adjusted to account for any differential non-response that may have occurred. Geodemographic distributions (age, gender, race, census region, and household income) for the corresponding youth and young adult populations were obtained from the March 2021 Supplement of the US Census Bureau’s Current Population Survey (CPS), the US Census Bureau’s American Community Survey (ACS), or from the weighted KnowledgePanel profile data. An iterative proportional fitting (raking) procedure was used to produce the final weights. Weights were examined to identify and, if necessary, trim outliers at the extreme upper and lower tails of the weight distribution. The resulting weights were then scaled to aggregate to the total sample size of all eligible respondents.

Prevalence estimates, including a weighted percentage and 95% confidence intervals (CI), were reported. Data were weighted using the effective base weight function. CIs were calculated using the Jeffreys method which is suitable for both large and small sample sizes [36]. Weighted prevalence estimates were used to calculate the estimated weighted number of persons and the 95% confidence interval based on the March 2021 US Census Bureau Current Population Survey. The estimated number of persons was calculated by dividing the population estimate by the weighed percentage then rounded down to the nearest 10,000 persons. All analyses were conducted in SPSS v27.0.

## Results

Results are presented below for the use of combustible cigarettes, e-cigarettes, the BIDI® Stick brand, and BIDI® Stick flavor variants by youth and underage young adults. Subgroup analysis was not possible as a result of levels of use being too low within these age cohorts.

### Use of combustible cigarettes and e-cigarettes

In Tables 1 and 2, we summarize the data on prevalence estimates for combustible cigarettes and e-cigarettes among youth and young adults, respectively.

**Table 1:** Prevalence estimates for combustible cigarettes and e-cigarettes among a probability-based sample of youth

*N*	13–17 years	
	1215	
	W% [95% CI]	EWNP [95% CI]^‡^
*Combustible cigarettes*		
Has ever smoked cigarettes^a,b^	11.15 [9.25–13.29]	2,350,000 [1,950,000–2,800,000]
Has smoked in the past 30 days^c,d^	3.50 [2.46–4.83]	730,000 [510,000–1,020,000]
*E-cigarettes*		
Is aware of e-cigarettes^e^	88.6% [86.4, 90.5]	18,720,000 [18,250,000–19,120,000]
Has ever used e-cigarettes^f,g^	14.64 [12.48–17.02]	3,090,000 [2,630,000–3,590,000]
Has used e-cigarettes in the past 30 days^c,h^	6.73 [5.26–8.48]	1,420,000 [1,110,000–1,790,000]

*N* unweighted number of participants, *W%* weighted percentage, *CI* confidence interval, and *EWNP* estimated weighted number of persons

^‡^Rounded down to the nearest 10,000 persons

^a^Even one or two puffs

^b^Question shown to all participants. Variable ID: [CIG1 = 1]

^c^On at least one of the past 30 days

^d^Question shown to participants who had ever smoked. Variable ID: [DNYTSSS = 1]

^e^Question shown to all participants. Variable ID: [ECI1]

^f^Even once or twice

^g^Question shown to participants who had seen or heard of e-cigarettes before this study. Variable ID: [ECI2 = 1]

^h^Question shown to participants who had ever used an e-cigarette. Variable ID: [DNYTSES = 1]

**Table 2:** Prevalence estimates for combustible cigarettes and e-cigarettes among a probability-based sample of young adults

*N*	18–20 years	
	1125	
	W% [95% CI]	EWNP [95% CI] ‡
*Combustible cigarettes*		
Has ever smoked cigarettes^a,b^	24.72 [21.18–28.54]	2,980,000 [2,550,000–3,440,000]
Now smokes every day or some days^c^	7.22 [5.24–9.67]	870,000 [630,000–1,160,000]
*E-cigarettes*		
Is aware of e-cigarettes^d^	89.3 [86.5–91.8]	10,770,000 [10,440,000–11,080,000]
Has ever used e-cigarettes^e,f^	34.62 [30.65–38.77]	4,170,000 [3,690,000–4,670,000]
Now uses e-cigarettes every day or some days^g^	15.90 [12.96–19.21]	1,910,000 [1,560,000–2,310,000]

*N* unweighted number of participants, *W%* weighted percentage, *CI* confidence interval, and *EWNP* estimated weighted number of persons

^‡^Rounded down to the nearest 10,000 persons

^a^Even one or two puffs

^b^Question shown to all participants. Variable ID: [CIG1 = 1]

^c^Question shown to participants who had ever smoked. Variable ID: [DESDSS = 1]

^d^Question shown to all participants. Variable ID: [ECI1 = 1]

^e^Even once or twice

^f^Question shown to participants who had seen or heard of e-cigarettes before this study. Variable ID: [ECI2 = 1]

^g^Question shown to participants who had ever used an e-cigarette. Variable ID: [DESDES = 1]

In Table 1, 11.15% of youth had ever smoked combustible cigarettes, and 3.50% had smoked combustible cigarettes in the past 30 days. In relation to e-cigarettes, 88.6% were aware of e-cigarettes, 14.64% reported having ever used e-cigarettes, and 6.73% reported using e-cigarettes in the past 30 days. In Table 2, 24.72% of young adults reported having ever smoked combustible cigarettes, and 7.22% reported smoking combustible cigarettes every day or some days. In relation to e-cigarettes, 89.3% were aware of e-cigarettes, 34.62% reported having ever used e-cigarettes, and 15.90% reported using e-cigarettes every day or some days.

From the estimates presented in Tables 1 and 2, the rate of current e-cigarette use among youth and young adults is almost double the rate of current smoking. Based on these data, it is easy to see why concern has been expressed at the extent of e-cigarette use among youth and young adults within the US, with some commentators fearing that the public health gains associated with the reduction in smoking in recent decades may be being diluted with the growth in the use of e-cigarettes and other novel nicotine delivery systems [37–39].

### Use of the BIDI® Stick brand

Tables 3 and 4 report data on the extent to which youth and young adults within the US were using the BIDI® Stick disposable e-cigarette device.

**Table 3:** Prevalence estimates for the BIDI® Stick brand among a probability-based sample of youth

	13–17 years		13–14 years		15–17 years	
	W% [95% CI]	EWNP [95% CI] ‡	W% [95% CI]	EWNP [95% CI] ‡	W% [95% CI]	EWNP [95% CI] ‡
Has ever used the brand^a^	0.91 [0.44–1.68]	190,000 [90,000–350,000]	0.32 [0.04–1.32]	20,000 [0000–100,000]	1.29 [0.58–2.51]	160,000 [70,000–320,000]
Has used the brand in the past 30 days^b^	0.04 [0.00–0.38]	< 10,000	0.00^†^	< 10,000	0.07 [0.00–0.63]	< 10,000

*W%* weighted percentage, *CI* confidence interval, and *EWNP* estimated weighted number of persons

^‡^Rounded down to the nearest 10,000 persons

^†^95% CI not reported

^a^Even once or twice

^b^On at least one of the past 30 days

**Table 4:** Prevalence estimates for the BIDI® Stick brand among a probability-based sample of young adults

	18–20 years	
	W% [95% CI]	EWNP [95% CI]^‡^
Has ever used the brand^a^	3.90 [2.49–5.81]	470,000 [300,000–700,000]
Now uses the brand every day or some days	0.60 [0.17–1.55]	70,000 [20,000–180,000]

*W%* weighted percentage, *CI* confidence interval, and *EWNP* estimated weighted number of persons

^‡^Rounded down to the nearest 10,000 persons

^a^Even once or twice

In Table 3, an estimated 0.91% or 190,000 youth reported having ever used a BIDI® Stick branded product. Less than 10,000 (0.04%) youth participants reported using the BIDI® Stick brand in the past 30 days. In Table 4, an estimated 3.90% or 470,000 young adults reported having ever used the BIDI® Stick brand. An estimated 70,000 (0.60%) young adults reported now using a BIDI® Stick branded product “every day” or “some days.”

### Use of BIDI® Stick flavor variants among youth

There were no youth participants who reported having ever used the BIDI® Stick in Winter, Marigold, and Dawn flavor variants [0.00%, < 10,000]. There were more youth who reported having ever used the tobacco flavor variant BIDI® Stick Classic [0.10%, 20,000]) than who reported having used the menthol flavor variant BIDI® Stick Arctic [0.08%, 10,000]. Youth participants reported ever using other BIDI® Stick flavor variants with the following frequencies: (Regal [0.02%, < 10,000]; Gold [0.06%, 10,000]; Tropic [0.08%, 10,000]; Solar [0.09%, 10,000]; Summer [0.13%, 20,000]; and Zest [0.25%, 50,000]). Use of a BIDI® Stick by youth in the past 30 days was reported for the BIDI® Stick Summer only [0.04%, < 10,000] (Table 5).

**Table 5:** Prevalence estimates for BIDI® Stick flavor variants among a probability-based sample of youth

	13–17 years		13–14 years		15–17 years	
	W% [95% CI]	EWNP [95% CI]‡	W% [95% CI]	EWNP [95% CI] ‡	W% [95% CI]	EWNP [95% CI] ‡
*BIDI*^*®*^* Stick Arctic*						
Has never used	99.50 [98.88–99.82]	21,020,000 [20,890,000–21,090,000]	99.79 [98.88–99.98]	8,270,000 [8,200,000–8,290,000]	99.31 [98.34–99.78]	12,740,000 [12,620,000–12,800,000]
Has ever used	0.08 [0.01–0.44]	10,000 [0000–90,000]	0.00^†^	< 10,000	0.13 [0.01–0.74]	10,000 [0000–90,000]
Has used in the past 30 days	0.00^†^	< 10,000	0.00^†^	< 10,000	0.00^†^	< 10,000
*BIDI*^*®*^* Stick Classic *						
Has never used	99.48 [98.85–99.80]	21,020,000 [20,880,000–21,080,000]	99.79 [98.88–99.98]	8,270,000 [8,200,000–8,290,000]	99.28 [98.28–99.76]	12,740,000 [12,610,000–12,800,000]
Has ever used	0.10 [0.01–0.49]	20,000 [0000–100,000]	0.00^†^	< 10,000	0.16 [0.02–0.81]	20,000 [0000–100,000]
Has used in the past 30 days	0.00^†^	< 10,000	0.00^†^	< 10,000	0.00^†^	< 10,000
*BIDI*^*®*^* Stick Zest*						
Has never used	99.33 [98.64–99.71]	20,980,000 [20,840,000–21,070,000]	99.79 [98.88–99.98]	8,270,000 [8,200,000–8,290,000]	99.03 [97.93–99.62]	12,710,000 [12,570,000–12,780,000]
Has ever used	0.25 [0.06–0.74]	50,000 [10,000–150,000]	0.00^†^	< 10,000	0.41 [0.09–1.24]	50,000 [10,000–150,000]
Has used in the past 30 days	0.00^†^	< 10,000	0.00^†^	< 10,000	0.00^†^	< 10,000
*BIDI*^*®*^* Stick Winter*						
Has never used	99.58 [98.99–99.86]	21,040,000 [20,910,000–21,100,000]	99.79 [98.88–99.98]	8,270,000 [8,200,000–8,290,000]	99.44 [98.53–99.84]	12,760,000 [12,640,000–12,810,000]
Has ever used	0.00^†^	< 10,000	0.00^†^	< 10,000	0.00^†^	< 10,000
Has used in the past 30 days	0.00^†^	< 10,000	0.00^†^	< 10,000	0.00^†^	< 10,000
*BIDI*^*®*^* Stick Tropic*						
Has never used	99.49 [98.87–99.81]	21,020,000 [20,890,000–21,090,000]	99.79 [98.88–99.98]	8,270,000 [8,200,000–8,290,000]	99.30 [98.32–99.77]	12,740,000 [12,620,000–12,800,000]
Has ever used	0.08 [0.01–0.45]	10,000 [0000–90,000]	0.00^†^	< 10,000	0.14 [0.01–0.76]	10,000 [0000–90,000]
Has used in the past 30 days	0.00^†^	< 10,000	0.00^†^	< 10,000	0.00^†^	< 10,000
*BIDI*^*®*^* Stick Gold*						
Has never used	99.52 [98.90–99.82]	21,020,000 [20,890,000–21,090,000]	99.68 [98.69–99.96]	8,260,000 [8,180,000–8,290,000]	99.41 [98.48–99.82]	12,760,000 [12,640,000–12,810,000]
Has ever used	0.06 [0.00–0.41]	10,000 [0000–80,000]	0.10 [0.00–0.90]	< 10,000	0.03 [0.00–0.54]	< 10,000
Has used in the past 30 days	0.00^†^	< 10,000	0.00^†^	< 10,000	0.00^†^	< 10,000
*BIDI*^*®*^* Stick Marigold*						
Has never used	99.58 [98.99–99.86]	21,040,000 [20,910,000–21,100,000]	99.79 [98.88–99.98]	8,270,000 [8,200,000–8,290,000]	99.44 [98.53–99.84]	12,760,000 [12,640,000–12,810,000]
Has ever used	0.00^†^	< 10,000	0.00^†^	< 10,000	0.00^†^	< 10,000
Has used in the past 30 days	0.00^†^	< 10,000	0.00^†^	< 10,000	0.00^†^	< 10,000
*BIDI*^*®*^* Stick Regal*						
Has never used	99.56 [98.96–99.85]	21,030,000 [20,910,000–21,090,000]	99.79 [98.88–99.98]	8,270,000 [8,200,000–8,290,000]	99.41 [98.48–99.82]	12,760,000 [12,640,000–12,810,000]
Has ever used	0.02 [0.00–0.32]	< 10,000	0.00^†^	< 10,000	0.03 [0.00–0.54]	< 10,000
Has used in the past 30 days	0.00^†^	< 10,000	0.00^†^	< 10,000	0.00^†^	< 10,000
*BIDI*^*®*^* Stick Summer*						
Has never used	99.45 [98.81–99.79]	21,010,000 [20,870,000–21,080,000]	99.79 [98.88–99.98]	8,270,000 [8,200,000–8,290,000]	99.23 [98.21–99.73]	12,730,000 [12,600,000–12,800,000]
Has ever used	0.13 [0.02–0.54]	20,000 [0000–110,000]	0.00^†^	< 10,000	0.21 [0.03–0.90]	20,000 [0000–110,000]
Has used in the past 30 days	0.04 [0.00–0.38]	< 10,000	0.00^†^	< 10,000	0.07 [0.00–0.63]	< 10,000
*BIDI*^*®*^* Stick Solar*						
Has never used	99.49 [98.86–99.81]	21,020,000 [20,890,000–21,090,000]	99.79 [98.88–99.98]	8,270,000 [8,200,000–8,290,000]	99.29 [98.30–99.76]	12,740,000 [12,610,000–12,800,000]
Has ever used	0.09 [0.01–0.47]	10,000 [0000–90,000]	0.00^†^	< 10,000	0.15 [0.01–0.79]	10,000 [0000–100,000]
Has used in the past 30 days	0.00^†^	< 10,000	0.00^†^	< 10,000	0.00^†^	< 10,000
*BIDI*^*®*^* Stick Dawn*						
Has never used	99.58 [98.99–99.86]	21,040,000 [20,910,000–21,100,000]	99.79 [98.88–99.98]	8,270,000 [8,200,000–8,290,000]	99.44 [98.53–99.84]	12,760,000 [12,640,000–12,810,000]
Has ever used	0.00^†^	< 10,000	0.00^†^	< 10,000	0.00^†^	< 10,000
Has used in the past 30 days	0.00^†^	< 10,000	0.00^†^	< 10,000	0.00^†^	< 10,000

*W%* weighted percentage, *CI* confidence interval, and *EWNP* estimated weighted number of people

^‡^Rounded down to the nearest 10,000 persons

^†^95% CI not reported

### Use of BIDI® Stick flavor variants among young adults

Fewer young adults reported using the tobacco flavored BIDI® Stick Classic (0.22%, 20,000) every day or some days compared to the menthol flavored BIDI® Stick Arctic (0.30%, 30,000). No young adults reported having used the BIDI Stick Classic 100 or more times in their lifetime and now using the product every day or some days, whereas 0.08% or < 10,000 young adults reported using the BIDI® Stick Arctic 100 or more times in their lifetime and now using the product every day or some days. Young adults reported using the BIDI® Stick Dawn [0.04%; < 10,000], Winter [0.04%; < 10,000], Zest [0.05%; < 10,000], Marigold [0.10%; < 10,000], Solar [0.15%; 10,000], Gold [0.25%; 30,000], and Tropic [0.27%; 30,000] every day or some days. Less than 10,000 young adults who reported having used each of these flavor variants every day or some days reported that they had used the flavor variant 100 or more times in their lifetime (Table 6).

**Table 6:** Prevalence estimates for BIDI® Stick flavor variants among a probability-based sample of young adults

	18–20 years	
	W% [95% CI]	EWNP [95% CI] ‡
*BIDI*^*®*^* Stick Arctic*		
Now uses every day or some days	0.30 [0.05–1.08]	30,000 [0000–130,000]
Has used 100 or more times	0.08 [0.00–0.67]	< 10,000
Has used less than 100 times	0.22 [0.03–0.93]	20,000 [0000–110,000]
*BIDI*^*®*^* Stick Classic *		
Now uses every day or some days	0.22 [0.03–0.95]	20,000 [0000–110,000]
Has used 100 or more times	0.00^†^	< 10,000
Has used less than 100 times	0.22 [0.03–0.95]	20,000 [0000–110,000]
*BIDI*^*®*^* Stick Zest*		
Now uses every day or some days	0.05 [0.00–0.61]	< 10,000
Has used 100 or more times	0.00^†^	< 10,000
Has used less than 100 times	0.05 [0.00–0.61]	< 10,000
*BIDI*^*®*^* Stick Winter*		
Now uses every day or some days	0.04 [0.00–0.57]	< 10,000
Has used 100 or more times	0.00^†^	< 10,000
Has used less than 100 times	0.04 [0.00–0.57]	< 10,000
*BIDI*^*®*^* Stick Tropic*		
Now uses every day or some days	0.27 [0.04–1.02]	30,000 [0000–120,000]
Has used 100 or more times	0.00^†^	< 10,000
Has used less than 100 times	0.27 [0.04–1.02]	30,000 [0000–120,000]
*BIDI*^*®*^* Stick Gold*		
Now uses every day or some days	0.25 [0.04–0.99]	30,000 [0000–110,000]
Has used 100 or more times	0.00^†^	< 10,000
Has used less than 100 times	0.25 [0.04–0.99]	30,000 [0000–110,000]
*BIDI*^*®*^* Stick Marigold*		
Now uses every day or some days	0.10 [0.01–0.71]	10,000 [0000–80,000]
Has used 100 or more times	0.00^†^	< 10,000
Has used less than 100 times	0.10 [0.01–0.71]	10,000 [0000–80,000]
*BIDI*^*®*^* Stick Regal*		
Now uses every day or some days	0.20 [0.02–0.91]	20,000 [0000–100,000]
Has used 100 or more times	0.08 [0.00–0.67]	< 10,000
Has used less than 100 times	0.12 [0.01–0.75]	10,000 [0000–90,000]
*BIDI*^*®*^* Stick Summer*		
Now uses every day or some days	0.14 [0.01–0.80]	10,000 [0000–90,000]
Has used 100 or more times	0.08 [0.00–0.67]	< 10,000
Has used less than 100 times	0.06 [0.00–0.62]	< 10,000
*BIDI*^*®*^* Stick Solar*		
Now uses every day or some days	0.15 [0.01–0.80]	10,000 [0000–90,000]
Has used 100 or more times	0.00^†^	< 10,000
Has used less than 100 times	0.15 [0.01–0.80]	10,000 [0000–90,000]
*BIDI*^*®*^* Stick Dawn*		
Now uses every day or some days	0.04 [0.00–0.58]	< 10,000
Has used 100 or more times	0.00^†^	< 10,000
Has used less than 100 times	0.04 [0.00–0.58]	< 10,000

*W%* weighted percentage, *CI* confidence interval, and *EWNP* estimated weighted number of people

^‡^Rounded down to the nearest 10,000 persons

^†^95% CI not reported

On the basis of these data, there is very little evidence of the BIDI® Stick product range being widely used by youth or underage young adults within the US. Similarly, while e-cigarette flavors have been widely presented as a driver of youth use of e-cigarettes within the US, in fact there was very little indication of the flavors available within the BIDI® Stick product range driving youth and underage young adult use of these products. In relation to current e-cigarette use among youth, there was reported use of only 1 of the 11 flavor variants of the BIDI® Stick, while among underage young adults, there were no flavor variants where the prevalence of current use was above 1.0%.

## Discussion

As well as documenting the extent of e-cigarette use by youth and underage young adults within the US, this paper has reported data on the extent to which these population groups were using the BIDI® Stick range of disposable flavored e-cigarettes. The finding of very low level of use of the BIDI® Stick disposable e-cigarettes among youth and underage young adults is important, in part, because of the tendency to view all disposable e-cigarettes as posing the same level of harm with regard to youth use.

Under the Premarket Tobacco Product Application (PMTA) process, the US Food and Drug Administration is seeking to determine whether individual e-cigarette products can be judged “appropriate for the protection of the public health,” and, on that basis, whether those products should be allowed to be sold within the US [40]. In making that determination regulators are seeking to weigh the evidence as to whether the specific products concerned are assisting adults who smoke in quitting, alongside the evidence of any public health harm where these products are being used (or are likely to be used) by vulnerable populations, including youth and those young adults below the legal age at which tobacco products can be purchased within the US. In weighing the potential benefit to adults who smoke against the harms to young people, it is clearly important to obtain information on both the degree to which individual e-cigarette products are indeed assisting adults who smoke in quitting and information on the extent to which the products concerned are being used by young people. However, as Morean and colleagues [41] have shown, simply asking young people about whether they have used an e-cigarette in general, rather than asking about specific e-cigarette devices they have used, may seriously underestimate levels of youth use.

It is important to emphasize here that the findings of very low levels of youth and underage young adult using the BIDI® Stick brand do not mean that this specific product range should be judged “appropriate for the protection of the public health” under the FDA’s PMTA process. Nevertheless, the evidence of such low levels of use would be consistent with a product that was judged appropriate for the protection of the public health where such data sit alongside evidence showing that the products were indeed assisting adults who smoke in quitting.

The importance of obtaining device-specific prevalence estimates of e-cigarette use among youth and underage adults is important in a broader context than the regulation of the products themselves. As has been shown with the experience of the JUUL e-cigarette, there is a tendency when it comes to e-cigarettes for a public narrative of harm to rapidly escalate and further for that narrative to focus upon a specific named device or, as in the case of disposable e-cigarettes, on an entire category of devices. Within such a context, calls to ban specific products or categories of products can rapidly gain momentum potentially impacting upon the likelihood that the product receives a marketing authorization, and the likelihood that the product will be used by those who are smoking combustible tobacco products. Within a context in which individual e-cigarette products become the focus of a stigmatizing narrative, there is also the very real possibility that those who are seen to be using these products will be subjected to stigmatizing comments, potentially further reducing the likelihood that these products will be used as a mean of quitting smoking.

Within a context in which there is a clear tendency to characterize specific e-cigarette devices, and categories of devices (as has occurred with disposable e-cigarettes), as being especially harmful, there is the risk of proscribing products that may also be assisting significant numbers of adults who smoke in quitting their use of combustible tobacco products. If e-cigarettes, and other non-combustion-based means of consuming nicotine, are to realize their potential as a means of reducing smoking-related health harm, there is a need to ensure that regulatory decisions determining which products are allowed to be sold are based on the best available evidence as to the extent to which those products are assisting adults who smoke in quitting, and the extent to which they are being used by young people and vulnerable groups. Ensuring that due weight is being given to such evidence within a context of heightened political rhetoric and concerted lobbying is unlikely to be an easy task.

There is a further challenge when it comes to determining which e-cigarette devices are, or are not, judged to be “appropriate for the protection of the public health” which is the apparent speed with which changes can occur within the e-cigarette market. Within the recent past, the JUUL e-cigarette has been characterized as one of the leading drivers of the epidemic in youth e-cigarette use within the US [42, 43]. Within the most recent National Youth Tobacco Survey, however, the JUUL device is no longer identified as one of the leading named brands used by youth within the US. The fact that an individual product can rapidly rise and fall in popularity further underlines the importance of ensuring that regularly updated information is available on the extent to which these devices are being used by diverse population groups [44].

Finally, findings of this study should be interpreted within the context of several limitations. Firstly, there was no pre-testing of participants’ comprehension of survey questions. However, such pre-testing was not deemed to be necessary as all questions and response options used in this study had been extracted or closely adapted from NYTS 2021 and the PATH Wave 5 Youth Interview Form, both of which were developed based on extensive cognitive testing and are well-established as comprehensible to an 8th grade reading level. Secondly, the estimates reported here are limited by a reliance on accurate, honest, self-reporting of tobacco product use behaviors. Self-reported tobacco product use may be subject to response bias. For the youth population, although participants were asked to complete and submit the survey in private and were assured that their answers would not be disclosed to their parents, participants may have been reluctant to report underage use of tobacco products. However, the validity of self-reported tobacco product use has, overall, been shown to be high in population-based studies [45]. To enhance the accuracy of self-reported brand and device usage data, this study combined both narrative questioning and image presentation both of the devices enquired about, and the brand images associated with each device. Showing an image of a tobacco product carries a risk of making a proportion of participants, especially youth, aware of e-cigarettes for the first time and increasing their curiosity to try using e-cigarettes. To mitigate this risk, participants were only shown a tobacco product image if they reported that they were aware of e-cigarettes before the study.

## Conclusions

The accumulated evidence that e-cigarettes are substantially less harmful than combustible cigarettes, and that these devices can assist adults who smoke in quitting, has underlined the capacity of these devices to substantially reduce smoking-related health harm. Whether these devices will be available to all of those who might benefit from their use will depend to a large extent on how e-cigarettes, and other non-combustion-based nicotine delivery systems, are regulated, and the extent to which those who are smoking choose to use these devices as an alternative to combustible tobacco products.

In the case of those countries that have chosen to ban these products such a regulatory decision effectively removes the option on the part of those who are smoking to use these devices as a means of reducing their exposure to significant health harm. However, even in those countries that have chosen to allow e-cigarettes to be available to those who are smoking, regulations can still be applied that limit individual’s access to specific products or specific categories of product. Restrictive regulatory actions initiated against disposable e-cigarettes are a case in point where the calls to regulatory intervention are underpinned by an assumption that all products within a category are presumed to have the same impact in terms of how widely they are being used by youth and underage young adults. As we have shown here, there are likely to be marked variations between products within the same category in terms of how widely they are being used by vulnerable populations.

To realize the harm reduction potential of e-cigarettes (including disposable e-cigarettes), it will be necessary to distinguish between products in terms of the extent to which they are being used by youth and underage young adults and the degree to which they are assisting adults who smoke in quitting. E-cigarette manufactures in general, and those associated with disposable e-cigarettes in particular, need to ensure that they have access to data showing the extent to which their specific products are being used by youth and underage young adults, and the extent to which their products are assisting adults who smoke to quit, in order to counter an emerging narrative which views all disposable e-cigarettes (and on occasion all e-cigarettes) as synonymous with public health harm.

### Supplementary Information

**Additional file 1:** Young adult survey instrument.

**Additional file 2:** Youth survey instrument.

## Data Availability

CSUR is currently analyzing data from their self-funded, multi-brand ENDS prevalence study, and as a result, it is not possible at the present time to make the data from this study available for wider analysis. CSUR is able to share the data gathering instrument used in this research for both youth and young adults.
