# Supplementary material for: When it comes to assessing the impact of e-cigarettes, estimates of device prevalence matter: the BIDI Stick disposable device
Source: Harm Reduct J. 2023 Jul 5;20:85. doi: 10.1186/s12954-023-00820-y (PMC10324178; doi:10.1186/s12954-023-00820-y)
Supplement: Supplementary file 1 — Additional file 1: Young adult survey instrument. [file 12954_2023_820_MOESM1_ESM.docx]

Base: all respondents

Sample type

1. KP
2. Off Panel

Base: all respondents

*Scripter: If consent is no or refused terminate*

**QUESTIONNAIRE**

**SAMPLE TYPE=2 DEMOS**

Base: all respondents (Sample type=1 or 2)

**[PPAGE]**

AGECONS [Q]

How old are you?

**[PROMPT]**

Your answer will help represent the entire U.S. population and will be kept confidential. Thank you!

*Type in your age.*

*SCRIPTER: min.=0, max.=120. Show label to right of box: years old. Prompt following nonresponse.*

*SCRIPTE: If age = 18 – 24 continue, all others terminate.*

Base: all respondents

**[PPGENDER]**

QGENDER [S]

Are you…?

**[PROMPT]**

Your answer will help represent the entire U.S. population and will be kept confidential. Thank you!

*Select one answer only.*

1. Male

2. Female

Base: all respondents

[PPSTATEN]

[PPREG4]

QSTATE [S]

In which state do you live?

**[PROMPT]**

We would like to have your answer to this question.

*Select one answer only.*

63. Alabama

94. Alaska

86. Arizona

71. Arkansas

93. California

84. Colorado

16. Connecticut

51. Delaware

53. District of Columbia

59. Florida

58. Georgia

95. Hawaii

82. Idaho

33. Illinois

32. Indiana

42. Iowa

47. Kansas

61. Kentucky

72. Louisiana

11. Maine

52. Maryland

14. Massachusetts

34. Michigan

41. Minnesota

64. Mississippi

43. Missouri

81. Montana

46. Nebraska

88. Nevada

12. New Hampshire

22. New Jersey

85. New Mexico

21. New York

56. North Carolina

44. North Dakota

31. Ohio

73. Oklahoma

92. Oregon

23. Pennsylvania

15. Rhode Island

57. South Carolina

45. South Dakota

62. Tennessee

74. Texas

87. Utah

13. Vermont

54. Virginia

91. Washington

55. West Virginia

35. Wisconsin

83. Wyoming

*SCRIPTER: Assign numeric codes per list above, but show full state name in alphabetic order in programmed survey. Show as drop down of all states and Washington DC.*

*Create data-only variables.*

**Variable name:** PPREG4

**Type:** SP

**Variable Text:**  Region 4 – based on State of residence

**Response list:**

1.  Northeast

2.  Midwest

3.  South

4.  West

| **PPSTATEN** | **PPREG4** |
| --- | --- |
| 11-23 | 1 |
| 31-47 | 2 |
| 51-74 | 3 |
| 81-95 | 4 |

Base: all respondents

[PPMSACAT]

QZIP [Q]

What is the ZIP Code where you live?

*SCRIPTER: min.=00000, max.=99999; require a 5-digit response.*

**Variable name:** QZIP

**Type:** SP

[Use crosswalk table based on zip_level_table. xlsx]

Base: all respondents

[PPEDUC]

[PPEDUCAT]

[PPEDUC5]

QEDUC [S]

What is the highest level of school you have completed?

**[PROMPT]**

We would like to have your answer to this question.

*Select one answer only.*

15. Some high school or less – no diploma or GED

9. High school graduate – high school diploma or the equivalent (GED)

10. Some college, no degree

11. Associate degree

12. Bachelor’s degree

13. Master’s degree

14. Professional or Doctorate degree

Base: respondents with some high school or less (QEDUC=15)

QEDUCa [S]

What is the highest level of school you have completed?

**[PROMPT]**

We would like to have your answer to this question.

*Select one answer only.*

1. No formal education

2. 1st, 2nd, 3rd, or 4th grade

3. 5th or 6th grade

4. 7th or 8th grade

5. 9th grade

6. 10th grade

7. 11th grade

8. 12th grade NO DIPLOMA

*SCRIPTER: Create Data-only variables.*

**Variable name:** PPEDUC [S]

**Variable Text:**  Education - categorical

**Response list:**

1.  No formal education

2.  1st, 2nd, 3rd or 4th grade

3.  5th or 6th grade

4.  7th or 8th grade

5. 9th grade

6. 10th grade

7. 11th grade

8. 12th grade NO DIPLOMA

9. HIGH SCHOOL GRADUATE – high school diploma or the equivalent (GED)

10. Some college, no degree

11. Associate degree

12. Bachelor’s degree

13. Master’s degree

14. Professional or Doctorate degree

| **QEDUC** | **QEDUCa** | **PPEDUC** |
| --- | --- | --- |
|  | 1 | 1 |
|  | 2 | 2 |
|  | 3 | 3 |
|  | 4 | 4 |
|  | 5 | 5 |
|  | 6 | 6 |
|  | 7 | 7 |
|  | 8 | 8 |
| 9 |  | 9 |
| 10 |  | 10 |
| 11 |  | 11 |
| 12 |  | 12 |
| 13 |  | 13 |
| 14 |  | 14 |

**Variable name:** PPEDUCAT [S]

**Variable Text:**  Education - categorical

**Response list:**

1.  Less than HS

2.  HS

3.  Some college

4.  Bachelor or higher

| **QEDUC** | **PPEDUCAT** |
| --- | --- |
| 15 | 1 |
| 9 | 2 |
| 10-11 | 3 |
| 12-14 | 4 |

**Variable name:** PPEDUC5 [S]

**Variable Text:**  Education - categorical

**Response list:**

1.  No high school diploma or GED

2.  High school graduate (high school diploma or the equivalent GED)

3.  Some college or Associate degree

4.  Bachelor’s degree

5. Master’s degree or above

| **QEDUC** | **PPEDUC5** |
| --- | --- |
| 15 | 1 |
| 9 | 2 |
| 10-11 | 3 |
| 12 | 4 |
| 13-14 | 5 |

Base: all respondents

**[PPHISPAN]**

QRACE1 [M]

Are you Spanish, Hispanic, or Latino?

**[PROMPT]**

Your answer will help represent the entire U.S. population and will be kept confidential. Thank you!

*Select all answers that apply.*

1. No, I am not [S]

2. Yes, Mexican, Mexican-American, Chicano

3. Yes, Puerto Rican

4. Yes, Cuban, Cuban American

8. Yes, other Spanish, Hispanic, or Latino group (Please specify, for example Argentinean, Colombian, Dominican, Nicaraguan, Salvadoran, Spaniard, and so on) [O]

Base: respondents who indicated multiple countries of origin (more than one response selected for QRACE1_2 to QRACE1_8)

QRACE1a [S]

Which group do you identify with most closely?

**[PROMPT]**

Your answer will help represent the entire U.S. population and will be kept confidential. Thank you!

*Select one answer only.*

*Show only response options selected in QRACE1:*

2. Mexican, Mexican-American, Chicano

3. Puerto Rican

4. Cuban, Cuban American

8. Other Spanish, Hispanic, or Latino group

*SCRIPTER: Prompt following nonresponse.*

*SCRIPTER: Create Data-only variable PPHISPAN by using the below logic involving responses to QRACE1 and QRACE1a.*

**Variable name:** PPHISPAN [S]

**Variable Text:**  Census Hispanicity

**Response list:**

1.  Non-Hispanic

2.  Mexican, Mexican American, Chicano

3.  Puerto Rican

4.  Cuban, Cuban American

5.  Other Spanish, Hispanic, or Latino group

Count numhispan=QRACE1_2 QRACE1_3 QRACE1_4 QRACE1_5 (1).

| **QRACE1** | **NUMHISPAN** | **QRACE1a** | **PPHISPAN** |
| --- | --- | --- | --- |
| 1 | - | - | 1 |
| 2 | 1 | - | 2 |
| 3 | 1 | - | 3 |
| 4 | 1 | - | 4 |
| 8 | 1 | - | 8 |
| Any value | >1 | 2 | 2 |
| Any value | >1 | 3 | 3 |
| Any value | >1 | 4 | 4 |
| Any value | >1 | 5 | 8 |
| Any value | >1 | Refused | Randomly assign to one of the values chosen in QRACE1 |

Base: all respondents

QRACE2INTRO

Please indicate what you consider your race to be. We appreciate your effort to describe your background using these U.S. Census Bureau categories.

*SCRIPTER: Show on same screen as CPSRACE.*

Base: all respondents

CPSRACE [M]

Please choose one or more **race(s)** that you consider yourself to be.

**[PROMPT]**

Your answer will help represent the entire U.S. population and will be kept confidential. Thank you!

*Select all answers that apply.*

1. White

2. Black or African American

3. American Indian or Alaska Native

4. Asian

5. Native Hawaiian or other Pacific Islander

6. Some other race [O]

Base: respondents who are Asian (CPSRACE=4)

CPSASIAN [M]

Which of the following Asian groups are you?

*Select all answers that apply.*

1. Asian Indian

2. Chinese

3. Filipino

4. Japanese

5. Korean

6. Vietnamese

7. Other Asian (Please specify, for example Hmong, Laotian, Thai, Pakistani, Cambodian, and so on) [O]

Base: respondents who are Native Hawaiian/Pacific Islander (CPSRACE=5)

CPSNHPI [M]

Which of the following Native Hawaiian or Other Pacific Islander groups are you?

*Select all answers that apply.*

1. Native Hawaiian

2. Guamanian or Chamorro

3. Samoan

4. Other Pacific Islander (Please specify, for example Fijian, Tongan, and so on) [O]

*SCRIPTER: Create Data-only variable PPETHM by using the below logic involving responses to QRACE1, CPSRACE, CPSASIAN and CPSNHPI.*

**Variable name:** PPETHM [S]

**Variable Text:**  Census Ethnicity demographic

**Response list:**

1.  White, Non-Hispanic

2.  Black, Non-Hispanic

3.  Other, Non-Hispanic

4.  Hispanic

5.  2+ Races, Non-Hispanic

Compute Asian=0.

Compute nhopi=0.

If CPSASIAN_1=1 or CPSASIAN _2=1 or CPSASIAN _3=1 or CPSASIAN _4=1 or CPSASIAN _5=1 or CPSASIAN _6=1 or CPSASIAN _7=1 asian=1.

If CPSNHPI _1=1 or CPSNHPI_2=1 or CPSNHPI_3=1 or CPSNHPI_4=1 nhopi=1.

Count numraces=CPSRACE_1 CPSRACE_2 CPSRACE_3 asian nhopi CPSRACE_6 (1).

| **QRACE1** | **CPSRACE/CPSASIAN/**  **CPSNHPI** | **PPETHM** |
| --- | --- | --- |
| 1 | CPSRACE_1=1 and numraces=1 | 1 |
| 1 | CPSRACE_2=1 and numraces=1 | 2 |
| 1 | (CPSRACE_3=1 OR  CPSRACE_4=1 OR  CPSRACE_5=1 OR  CPSRACE_6=1)  and numraces=1 | 3 |
| 1 | numraces > 1 | 5 |
| 2 OR 3 OR 4 OR 8 | (numraces=1 or numraces>1) | 4 |
| REFUSED | Any value | MISSING |
| Any value | REFUSED | MISSING |
| 2 OR 3 OR 4 OR 8 | REFUSED | 4 |

**Variable name:** PPRACEM [S]

**Variable Text:**  Census Race demographic

**Response list:**

1.  White

2.  Black or African American

3. American Indian or Alaska Native

4. Asian

5.  Native Hawaiian/Pacific Islander

6.  2+ Races

| **CPSRACE/CPSASIAN/**  **CPSNHPI** | **PPRACEM** |
| --- | --- |
| CPSRACE_1=1 and numraces=1 | 1 |
| CPSRACE_2=1 and numraces=1 | 2 |
| CPSRACE_3=1 and numraces=1 | 3 |
| CPSRACE_4=1  OR and numraces=1 | 4 |
| CPSRACE_5=1)  and  numraces=1 | 5 |
| (numraces>1) | 6 |
| Any value | MISSING |
| REFUSED | MISSING |

Base: all respondents

[PPINCIMP]

QINC [S]

How much is the combined income of all members of YOUR HOUSEHOLD for the PAST 12 MONTHS?

**[SPACE]**

Please include your income PLUS the income of all members living in your household (including cohabiting partners and armed forces members living at home). Please count income BEFORE TAXES and from all sources (such as wages, salaries, tips, net income from a business, interest, dividends, child support, alimony, and Social Security, public assistance, pensions, or retirement benefits).

*Select one answer only.*

1. Below $50,000

2. $50,000 or more

3. Don’t know

*SCRIPTER: Prompt once if question is skipped. Do not show ‘Don’t know’ initially. Show ‘Don’t know’ only with the prompt if question is skipped initially.*

**[PROMPT]**

Your answer will help represent the entire U.S. population and will be kept confidential. Thank you!

Base: respondents with household income below $50,000 (QINC=1)

QINC2 [S]

We would like to get a better estimate of your total HOUSEHOLD income in the past 12 months before taxes. Was it...

**[PROMPT]**

Your answer will help represent the entire U.S. population and will be kept confidential. Thank you!

*Select one answer only.*

1. Less than $5,000

2. $5,000 to $7,499

3. $7,500 to $9,999

4. $10,000 to $12,499

5. $12,500 to $14,999

6. $15,000 to $19,999

7. $20,000 to $24,999

8. $25,000 to $29,999

9. $30,000 to $34,999

10. $35,000 to $39,999

11. $40,000 to $49,999

Base: respondents with household income of $50,000 or more (QINC=2)

QINC3 [S]

We would like to get a better estimate of your total HOUSEHOLD income in the past 12 months before taxes. Was it...

**[PROMPT]**

Your answer will help represent the entire U.S. population and will be kept confidential. Thank you!

*Select one answer only.*

3. $50,000 to $59,999

4. $60,000 to $74,999

5. $75,000 to $84,999

6. $85,000 to $99,999

7. $100,000 to $124,999

8. $125,000 to $149,999

9. $150,000 to $174,999

10. $175,000 to $199,999

11. $200,000 to $249,999

12. $250,000 or more

*SCRIPTER: Create Data-only variables below.*

**Variable name:** PPINC7 [S]

**Variable Text:**  HH income

**Response list:**

1 = Under $10,000

2 = $10,000 to $24,999

3 = $25,000 to $49,999

4 = $50,000 to $74,999

5 = $75,000 to $99,999

6 = $100,000 to $149,999

7 = $150,000 or more

| **QINC2** | **QINC3** | **PPINC7** |
| --- | --- | --- |
| 1,2,3 |  | 1 |
| 4,5,6,7 |  | 2 |
| 8,9,10,11 |  | 3 |
|  | 3,4 | 4 |
|  | 5,6 | 5 |
|  | 7,8 | 6 |
|  | 9,10,11,12 | 7 |

Base: all respondents

[PPMARIT5]

QMARIT [S]

Are you now...?

*Select one answer only.*

1. Married

2. Widowed

3. Divorced

4. Separated

5. Never married

*SCRIPTER: Create Data-only variable.*

**Variable name:** PPMARIT5 [S]

**Variable Text:**  Marital Status

**Response list:**

1. Now married

2. Widowed

3. Divorced

4. Separated

5. Never married

| **QMARIT** | **PPMARIT5** |
| --- | --- |
| 1 | 1 |
| 2 | 2 |
| 3 | 3 |
| 4 | 4 |
| 5 | 5 |

Base: all respondents

[PPT18OV]

QHHSIZE_adults [Q]

Including yourself, how many people are 18 years of age or older and currently live in your household at least 50% of the time?

**[SPACE]**

Please include unrelated individuals (such as roommates), and also include those now away traveling, away at school, or in a hospital.

**[PROMPT]**

Your answer will help represent the entire U.S. population and will be kept confidential. Thank you!

*Type in the number of adults 18 years of age or older.*

*SCRIPTER: min.=1, max.=10. Prompt following nonresponse. Show on same screen as Q5b.*

Base: all respondents

[PPKID017]

QHHSIZE_kids [Q]

Next, how many people are 17 years of age or younger and currently live in your household at least 50% of the time? If none, enter “0”.

**[SPACE]**

Include babies and small children.

**[PROMPT]**

Your answer will help represent the entire U.S. population and will be kept confidential. Thank you!

*Type in the number of children 17 years of age or younger.*

*SCRIPTER: min.=0, max.=10. Prompt following nonresponse.*

*SCRIPTER: Create Data-only variable.*

Compute QHHSIZE_kids = PPKID017.

**Variable name:** PPKID017

**Type:** Numeric

**Variable Text:**  Household Members Age 0 to 17

**Numeric range: 0-10 or 99**

Base: all respondents

[PPHHSIZE]

QHHSIZE [Q]

*SCRIPTER: Create DOV: QHHSIZE=QHHSIZE_adults + QHHSIZE_kids. Compute if QHHSIZE_adults and QHHSIZE_kids are not refused.*

Base: all respondents

[PPEMPLOY]

QEMPLOY [Q]

How many hours do you usually work for pay or profit per week? Please include hours you work for pay or profit at all your jobs if you have more than one job. If none, enter “0”. If less than an hour in a week, enter “1”.

__ __ __

*SCRIPTER: min.=0, max.=168. Show label to right of box: Hours per week. Do not allow decimals. Prompt following nonresponse. Create data only variable.*

IF QEMPLOY ≥ 35 PPEMPLOY = 1.

IF QEMPLOY ≤ 34 AND QEMPLOY ≥ 1 PPEMPLOY = 2.

IF QEMPLOY = 0 PPEMPLOY = 3.

**Variable name:** PPEMPLOY [S]

**Variable Text:**  Current employment status

**Response list:**

1. Working full-time

2. Working part-time

3. Not working

Base: all respondents

[PPRENT]

QOWN [S]

Are your living quarters…

*Select one answer only.*

1. Owned by you or someone in your household with a mortgage or loan

4. Owned by you or someone in your household free and clear (without a mortgage or loan)

2. Rented

3. Occupied without payment of rent

*SCRIPTER: Create Data-only variable.*

**Variable name:** PPRENT

**Type:** SP

**Variable Text:**  Home is owned or rented

| **QOWN** | **PPRENT** |
| --- | --- |
| 1, 4 | 1 |
| 2 | 2 |
| 3 | 3 |

Base: all respondents

[PPHOUSE4]

QHOUSE [S]

Which best describes the building where you live?

*Select one answer only.*

1. One-family house detached from any other house

2. One-family house attached to one or more houses (such as a condo or townhouse)

3. Building with 2 or more apartments

4. Mobile home

5. Boat, RV, van, etc.

*SCRIPTER: Create Data-only variable.*

**Variable name:** PPHOUSE4

**Type:** SP

**Variable Text:** Housing Type

1 = One-family house detached from any other house

2 = One-family condo or townhouse attached to other units

3 = Building with 2 or more apartments

4 = Other (mobile home, boat, RV, van, etc.)

| **QHOUSE** | **PPHOUSE4** |
| --- | --- |
| 1 | 1 |
| 2 | 2 |
| 3 | 3 |
| 4, 5 | 4 |

**1. COMBUSTIBLE CIGARETTES**

Base: all respondents

CIGINT [Descriptor Text]

The first questions are about smoking cigarettes (ones that have to be lit and burned).

Base: all respondents

CIG1 [S]

Have you ever tried cigarette smoking, even one or two puffs?

1. Yes

2. No

SCRIPTER: REQUEST RESPONSE

Base: if CIG1 = 1

CIG2 [O]

How old were you when you **first tried** cigarette smoking, even one or two puffs?

1. [SCRIPTER INSERT NUMERICAL ENTRY BOX] years old

CONTROL: ENTERED RESPONSE ≤ Age

SCRIPTER: INSERT NUMERICAL ENTRY BOX INTO RESPONSE OPTION 1, NO DECIMALS

Base: CIG1 = 1

CIG3B [S]

Do you now smoke cigarettes…

1. Every day

2. Some days

3. Not at all

SCRIPTER: REQUEST RESPONSE

Base: if CIG1 = 1

CIG3 [S]

How many cigarettes have you smoked in your **entire life**? A pack usually has 20 cigarettes in it.

1. 1 or more puffs but never a whole cigarette

2. 1 cigarette

3. 2 to 5 cigarettes

4. 6 to 15 cigarettes (about 1/2 a pack total)

5. 16 to 25 cigarettes (about 1 pack total)

6. 26 to 99 cigarettes (more than 1 pack, but less than 5 packs)

7. 100 or more cigarettes (5 or more packs)

SCRIPTER: REQUEST RESPONSE

Base: if CIG1 = 1

CIG4 [O]

In the **past 30 days**, on how many days did you smoke cigarettes?

1. [ENTER NUMBER] Day(s)

CONTROL:

Min: 0

Max: 30

SCRIPTER: INSERT NUMERICAL ENTRY BOX INTO RESPONSE OPTION 1, NO DECIMALS

Base: if CIG1 = 1

CIG5 [S]

When was the **last time** you smoked a cigarette, even one or two puffs?

1. Earlier today

2. Not today but sometime during the past 7 days

3. Not during the past 7 days but sometime during the past 30 days

4. Not during the past 30 days but sometime during the past 6 months

5. Not during the past 6 months but sometime during the past year

6. 1 to 4 years ago

7. 5 or more years ago

SCRIPTER: DO NOT DISPLAY RESPONSE OPTIONS 4 AND 5 AND 6 AND 7 if CIG4 ≥ 1

DO NOT DISPLAY RESPONSE OPTIONS 1 AND 2 AND 3 if CIG4 = 0

Base: CIG4 ≥ 1

CIG6 [S]

In the past 30 days, **on the days you smoked**, how many cigarettes did you smoke per day? A pack usually has 20 cigarettes in it.

1. Less than 1 cigarette per day

2. 1 cigarette per day

3. 2 to 5 cigarettes per day

4. 6 to 10 cigarettes per day

5. 11 to 20 cigarettes per day

6. More than 20 cigarettes per day

Base: CIG3=7 AND CIG3B=1 OR 2

CIG7 [S]

Are you seriously thinking about quitting **cigarettes**? (Please choose the first answer that fits)

1. Yes, during the next 30 days

2. Yes, during the next 6 months

3. Yes, during the next 12 months

4. Yes, but not during the next 12 months

5. No, I am not thinking about quitting cigarettes

Base: if CIG1 = 2

CIG8 [S]

Have you ever been curious about smoking a cigarette?

1. Very curious

2. Somewhat curious

3. A little curious

4. Not at all curious

998. DON’T KNOW

Base: if CIG1 = 2

CIG9 [S]

Do you think you will smoke a cigarette in the next year?

1. Definitely yes

2. Probably yes

3. Probably not

4. Definitely not

998. DON’T KNOW

Base: if CIG1 = 2

CIG10 [S]

Do you think that you will try a cigarette soon?

1. Definitely yes

2. Probably yes

3. Probably not

4. Definitely not

998. DON’T KNOW

Base: if CIG1 = 2

CIG11 [S]

If one of your best friends were to offer you a cigarette, would you smoke it?

1. Definitely yes

2. Probably yes

3. Probably not

4. Definitely not

998. DON’T KNOW

**2. GENERIC E-CIGARETTES**

Base: all respondents

ECIG_INT [Descriptor text]

The next several questions are about electronic cigarettes or e-cigarettes, such as JUUL, Vuse, blu, and Logic.. E-cigarettes are battery powered devices that usually contain a nicotine-based liquid that is vaporized and inhaled. You may also know them as e-cigs, vape-pens, e-hookahs, vapes, or mods.

Base: all respondents

ECI1 [S]

Have you ever seen or heard of e-cigarettes before this study?

1. Yes

2. No

SCRIPTER: REQUEST RESPONSE

Base: ECI1 = 1

ECI2 [S]

Have you **ever used** an e-cigarette, even one or two times?

1. Yes

2. No

SCRIPTER: REQUEST RESPONSE

Base: if ECI2 = 1

ECI3 [O]

How old were you when you **first used** an e-cigarette, even once or twice?

1. [SCRIPTER INSERT NUMERICAL ENTRY BOX] years old

CONTROL: ENTERED RESPONSE ≤ Age

SCRIPTER: INSERT NUMERICAL ENTRY BOX INTO RESPONSE OPTION 1, NO DECIMALS

Base: ECI2 = 1

ECI351 [S]

Do you now use e-cigarettes…

1. Every day

2. Some days

3. Not at all

SCRIPTER: REQUEST RESPONSE

Base: if ECI2 = 1

ECI4 [S]

How many times have you used an e-cigarette in your **entire life**?

1. 1 time, even just a few puffs

2. 2 to 10 times

3. 11 to 20 times

4. 21 to 50 times

5. 51 to 99 times

6. 100 or more times

SCRIPTER: REQUEST RESPONSE

Base: if ECI2 = 1

ECI5 [S]

During the **past 30 days**, on how many days did you use an e-cigarette?

1. [ENTER NUMBER] Day(s)

Min: 0

Max: 30

SCRIPTER: INSERT NUMERICAL ENTRY BOX TO RESPONSE OPTION 1, NO DECIMALS

Base: if ECI2 = 1

ECI6 [S]

When was the **last time** you used an e-cigarette, even one or two puffs? (Please choose the first answer that fits)

1. Earlier today

2. Not today but sometime during the past 7 days

3. Not during the past 7 days but sometime during the past 30 days

4. Not during the past 30 days but sometime during the past 6 months

5. Not during the past 6 months but sometime during the past year

6. 1 to 4 years ago

7. 5 or more years ago

SCRIPTER: DO NOT DISPLAY RESPONSE OPTIONS 4 AND 5 AND 6 AND 7 if ECI5 ≥ 1

DO NOT DISPLAY RESPONSE OPTIONS 1 AND 2 AND 3 if ECI5 = 0

Base: if ECI5 ≥ 1

[M]

Which flavors of e-liquid have you used in an e-cigarette in **the past 30 days**? Choose all that apply

ECIF1. Tobacco

ECIF2. Menthol/Mint

ECIF3. Fruit

ECIF4. Dessert (such as Muffin, Ice cream, custard or other desserts)

ECIF5. Alcoholic drink (such as bourbon, piña colada, mojito or other alcoholic drinks)

ECIF6. Other beverages (such as cola, energy drink, milk or other non-alcoholic beverages)

ECIF7. Candy (such as bubble gum, cotton candy, gummy bears or other candy)

ECIF8. Other sweets (such as chocolate, caramel, vanilla or other sweets)

ECIF9. Coffee/Tea (such as caffè latte, cappuccino, tea or other)
ECIF10. Spices (such as cinnamon, clove, licorice or other spices)

ECIF11. Nuts (such as hazelnut, peanut (butter), pecan or other nuts)
ECIF12. Another flavor not listed here [Specify: SCRIPTER INSERT TEXT BOX]

ECIF998. I DON’T KNOW

SCRIPTER: RESPONSE OPTION ECIF998 IS EXCLUSIVE

Base: if ECI2 = 1

[M] (Randomize)

Please look carefully at the brand logos below

Have you ever used any of these brands of e-cigarettes, even once or twice? (**Select all that you have ever used**)

ECI8265. [INSERT IMG_265]

ECI80. I have not used any of these brands of e-cigarettes

SCRIPTER: ANCHOR RESPONSE OPTION ECI80 AND MAKE EXCLUSIVE

Base: if ECI2 = 2

ECI11 [S]

Have you ever been curious about using an e-cigarette?

1. Very curious

2. Somewhat curious

3. A little curious

4. Not at all curious

998. DON’T KNOW

Base: if ECI2 = 2

ECI12 [S]

Do you think you will use an e-cigarette in the next year?

1. Definitely yes

2. Probably yes

3. Probably not

4. Definitely not

998. DON’T KNOW

Base: if ECI2 = 2

ECI13 [S]

Do you think that you will try an e-cigarette soon?

1. Definitely yes

2. Probably yes

3. Probably not

4. Definitely not

998. DON’T KNOW

Base: if ECI2 = 2

ECI14 [S]

If one of your best friends were to offer you an e-cigarette, would you use it?

1. Definitely yes

2. Probably yes

3. Probably not

4. Definitely not

998. DON’T KNOW

Base: ECI4=6 AND ECI351=1 OR 2

ECI15 [S]

Are you seriously thinking about quitting **e-cigarettes**?

1. Yes, during the next 30 days

2. Yes, during the next 6 months

3. Yes, during the next 12 months

4. Yes, but not during the next 12 months

5. No, I am not seriously thinking about quitting

Base: IF ECI8265 = 1

ECIINT2 [Descriptor text]

The next few questions will ask you about your use of specific e-cigarette devices.

In answering these questions, please ignore the device color. Please focus only on the device itself.

**EVER USE – BIDI® Stick**

Base: if ECI8265 = 1

[M] (Randomize)

You said that you have used a **BIDI® Stick** e-cigarette.

Below are the pictures of different e-cigarettes that are made by **BIDI^®^ Stick**,

Which of these **BIDI® Stick** e-cigarettes have you ever used, even once or twice? (Check all that apply)

BID281. [INSERT IMG_281]

BID282. [INSERT IMG_282]

BID283. [INSERT IMG_283]

BID284. [INSERT IMG_284]

BID285. [INSERT IMG_285]

BID286. [INSERT IMG_286]

BID287. [INSERT IMG_287]

BID288. [INSERT IMG_288]

BID289. [INSERT IMG_289]

BID290. [INSERT IMG_290]

BID291. [INSERT IMG_291]

BID0. I have not used any of these e-cigarettes

SCRIPTER: ANCHOR RESPONSE OPTION BID0 AND MAKE EXCLUSIVE

Base: BID281 = 1

BID281C [S]

[INSERT IMG_281]

Do you now use the **BIDI® Stick Arctic (**previously known as **BIDI® Stick Mint Freeze)…**

1. Every day

2. Some days

3. Not at all

SCRIPTER: IF ECI351 = 2, DO NOT DISPLAY OPTION 1

SCRIPTER: IF ECI351 = 3, AUTOFILL WITH 3 AND CONTINUE

Base: if BID281 = 1

BID281LT [S]

[INSERT IMG_281]

How many times have you used the **BIDI® Stick Arctic (**previously known as **BIDI® Stick Mint Freeze)** in your entire life?

1. 1 time, even just a few puffs

2. 2 to 10 times

3. 11 to 20 times

4. 21 to 50 times

5. 51 to 99 times

6. 100 or more times

SCRIPTER: ONLY DISPLAY RESPONSE OPTION 2 IF ECI4 = 2 OR 3 OR 4 OR 5 OR 6

ONLY DISPLAY RESPONSE OPTION 3 IF ECI4 = 3 OR 4 OR 5 OR 6

ONLY DISPLAY RESPONSE OPTION 4 IF ECI4 = 4 OR 5 OR 6

ONLY DISPLAY RESPONSE OPTION 5 IF ECI4 = 5 OR 6

ONLY DISPLAY RESPONSE OPTION 6 IF ECI4 = 6

Base: BID282 = 1

BID282C [S]

[INSERT IMG_282]

Do you now use the **BIDI® Stick Classic (**previously known as **BIDI® Stick Classic Tobacco)…**

1. Every day

2. Some days

3. Not at all

SCRIPTER: IF ECI351 = 2, DO NOT DISPLAY OPTION 1

SCRIPTER: IF ECI351 = 3, AUTOFILL WITH 3 AND CONTINUE

Base: if BID282 = 1

BID282LT [S]

[INSERT IMG_282]

How many times have you used the **BIDI® Stick Classic (**previously known as **BIDI® Stick Classic Tobacco)** in your entire life?

1. 1 time, even just a few puffs

2. 2 to 10 times

3. 11 to 20 times

4. 21 to 50 times

5. 51 to 99 times

6. 100 or more times

SCRIPTER: ONLY DISPLAY RESPONSE OPTION 2 IF ECI4 = 2 OR 3 OR 4 OR 5 OR 6

ONLY DISPLAY RESPONSE OPTION 3 IF ECI4 = 3 OR 4 OR 5 OR 6

ONLY DISPLAY RESPONSE OPTION 4 IF ECI4 = 4 OR 5 OR 6

ONLY DISPLAY RESPONSE OPTION 5 IF ECI4 = 5 OR 6

ONLY DISPLAY RESPONSE OPTION 6 IF ECI4 = 6

Base: BID283 = 1

BID283C [S]

[INSERT IMG_283]

Do you now use the **BIDI® Stick Zest (**previously known as **BIDI® Stick Jungle Juice)…**

1. Every day

2. Some days

3. Not at all

SCRIPTER: IF ECI351 = 2, DO NOT DISPLAY OPTION 1

SCRIPTER: IF ECI351 = 3, AUTOFILL WITH 3 AND CONTINUE

Base: if BID283 = 1

BID283LT [S]

[INSERT IMG_283]

How many times have you used the **BIDI® Stick Zest (**previously known as **BIDI® Stick Jungle Juice)** in your entire life?

1. 1 time, even just a few puffs

2. 2 to 10 times

3. 11 to 20 times

4. 21 to 50 times

5. 51 to 99 times

6. 100 or more times

SCRIPTER: ONLY DISPLAY RESPONSE OPTION 2 IF ECI4 = 2 OR 3 OR 4 OR 5 OR 6

ONLY DISPLAY RESPONSE OPTION 3 IF ECI4 = 3 OR 4 OR 5 OR 6

ONLY DISPLAY RESPONSE OPTION 4 IF ECI4 = 4 OR 5 OR 6

ONLY DISPLAY RESPONSE OPTION 5 IF ECI4 = 5 OR 6

ONLY DISPLAY RESPONSE OPTION 6 IF ECI4 = 6

Base: BID284 = 1

BID284C [S]

[INSERT IMG_284]

Do you now use the **BIDI® Stick Winter (**previously known as **BIDI® Stick Lush Ice)…**

1. Every day

2. Some days

3. Not at all

SCRIPTER: IF ECI351 = 2, DO NOT DISPLAY OPTION 1

SCRIPTER: IF ECI351 = 3, AUTOFILL WITH 3 AND CONTINUE

Base: if BID284 = 1

BID284LT [S]

[INSERT IMG_284]

How many times have you used the **BIDI® Stick Winter (**previously known as **BIDI® Stick Lush Ice)** in your entire life?

1. 1 time, even just a few puffs

2. 2 to 10 times

3. 11 to 20 times

4. 21 to 50 times

5. 51 to 99 times

6. 100 or more times

SCRIPTER: ONLY DISPLAY RESPONSE OPTION 2 IF ECI4 = 2 OR 3 OR 4 OR 5 OR 6

ONLY DISPLAY RESPONSE OPTION 3 IF ECI4 = 3 OR 4 OR 5 OR 6

ONLY DISPLAY RESPONSE OPTION 4 IF ECI4 = 4 OR 5 OR 6

ONLY DISPLAY RESPONSE OPTION 5 IF ECI4 = 5 OR 6

ONLY DISPLAY RESPONSE OPTION 6 IF ECI4 = 6

Base: BID285 = 1

BID285C [S]

[INSERT IMG_285]

Do you now use the **BIDI® Stick Tropic (**previously known as **BIDI® Stick Blazing Vibe)…**

1. Every day

2. Some days

3. Not at all

SCRIPTER: IF ECI351 = 2, DO NOT DISPLAY OPTION 1

SCRIPTER: IF ECI351 = 3, AUTOFILL WITH 3 AND CONTINUE

Base: if BID285 = 1

BID285LT [S]

[INSERT IMG_285]

How many times have you used the **BIDI® Stick Tropic (**previously known as **BIDI® Stick Blazing Vibe)** in your entire life?

1. 1 time, even just a few puffs

2. 2 to 10 times

3. 11 to 20 times

4. 21 to 50 times

5. 51 to 99 times

6. 100 or more times

SCRIPTER: ONLY DISPLAY RESPONSE OPTION 2 IF ECI4 = 2 OR 3 OR 4 OR 5 OR 6

ONLY DISPLAY RESPONSE OPTION 3 IF ECI4 = 3 OR 4 OR 5 OR 6

ONLY DISPLAY RESPONSE OPTION 4 IF ECI4 = 4 OR 5 OR 6

ONLY DISPLAY RESPONSE OPTION 5 IF ECI4 = 5 OR 6

ONLY DISPLAY RESPONSE OPTION 6 IF ECI4 = 6

Base: BID286 = 1

BID286C [S]

[INSERT IMG_286]

Do you now use the **BIDI® Stick Gold (**previously known as **BIDI® Stick Fruity Mango)…**

1. Every day

2. Some days

3. Not at all

SCRIPTER: IF ECI351 = 2, DO NOT DISPLAY OPTION 1

SCRIPTER: IF ECI351 = 3, AUTOFILL WITH 3 AND CONTINUE

Base: if BID286 = 1

BID286LT [S]

[INSERT IMG_286]

How many times have you used the **BIDI® Stick Gold (**previously known as **BIDI® Stick Fruity Mango)** in your entire life?

1. 1 time, even just a few puffs

2. 2 to 10 times

3. 11 to 20 times

4. 21 to 50 times

5. 51 to 99 times

6. 100 or more times

SCRIPTER: ONLY DISPLAY RESPONSE OPTION 2 IF ECI4 = 2 OR 3 OR 4 OR 5 OR 6

ONLY DISPLAY RESPONSE OPTION 3 IF ECI4 = 3 OR 4 OR 5 OR 6

ONLY DISPLAY RESPONSE OPTION 4 IF ECI4 = 4 OR 5 OR 6

ONLY DISPLAY RESPONSE OPTION 5 IF ECI4 = 5 OR 6

ONLY DISPLAY RESPONSE OPTION 6 IF ECI4 = 6

Base: BID287 = 1

BID287C [S]

[INSERT IMG_287]

Do you now use the **BIDI® Stick Marigold (**previously known as **BIDI® Stick Icy Mango)…**

1. Every day

2. Some days

3. Not at all

SCRIPTER: IF ECI351 = 2, DO NOT DISPLAY OPTION 1

SCRIPTER: IF ECI351 = 3, AUTOFILL WITH 3 AND CONTINUE

Base: if BID287 = 1

BID287LT [S]

[INSERT IMG_287]

How many times have you used the **BIDI® Stick Marigold (**previously known as **BIDI® Stick Icy Mango)** in your entire life?

1. 1 time, even just a few puffs

2. 2 to 10 times

3. 11 to 20 times

4. 21 to 50 times

5. 51 to 99 times

6. 100 or more times

SCRIPTER: ONLY DISPLAY RESPONSE OPTION 2 IF ECI4 = 2 OR 3 OR 4 OR 5 OR 6

ONLY DISPLAY RESPONSE OPTION 3 IF ECI4 = 3 OR 4 OR 5 OR 6

ONLY DISPLAY RESPONSE OPTION 4 IF ECI4 = 4 OR 5 OR 6

ONLY DISPLAY RESPONSE OPTION 5 IF ECI4 = 5 OR 6

ONLY DISPLAY RESPONSE OPTION 6 IF ECI4 = 6

Base: BID288 = 1

BID288C [S]

[INSERT IMG_288]

Do you now use the **BIDI® Stick Regal (**previously known as **BIDI® Stick Dragon Venom)…**

1. Every day

2. Some days

3. Not at all

SCRIPTER: IF ECI351 = 2, DO NOT DISPLAY OPTION 1

SCRIPTER: IF ECI351 = 3, AUTOFILL WITH 3 AND CONTINUE

Base: if BID288 = 1

BID288LT [S]

[INSERT IMG_288]

How many times have you used the **BIDI® Stick Regal (**previously known as **BIDI® Stick Dragon Venom)** in your entire life?

1. 1 time, even just a few puffs

2. 2 to 10 times

3. 11 to 20 times

4. 21 to 50 times

5. 51 to 99 times

6. 100 or more times

SCRIPTER: ONLY DISPLAY RESPONSE OPTION 2 IF ECI4 = 2 OR 3 OR 4 OR 5 OR 6

ONLY DISPLAY RESPONSE OPTION 3 IF ECI4 = 3 OR 4 OR 5 OR 6

ONLY DISPLAY RESPONSE OPTION 4 IF ECI4 = 4 OR 5 OR 6

ONLY DISPLAY RESPONSE OPTION 5 IF ECI4 = 5 OR 6

ONLY DISPLAY RESPONSE OPTION 6 IF ECI4 = 6

Base: BID289 = 1

BID289C [S]

[INSERT IMG_289]

Do you now use the **BIDI® Stick Summer (**previously known as **BIDI® Stick Kick Start)…**

1. Every day

2. Some days

3. Not at all

SCRIPTER: IF ECI351 = 2, DO NOT DISPLAY OPTION 1

SCRIPTER: IF ECI351 = 3, AUTOFILL WITH 3 AND CONTINUE

Base: if BID289 = 1

BID289LT [S]

[INSERT IMG_289]

How many times have you used the **BIDI® Stick Summer (**previously known as **BIDI® Stick Kick Start)** in your entire life?

1. 1 time, even just a few puffs

2. 2 to 10 times

3. 11 to 20 times

4. 21 to 50 times

5. 51 to 99 times

6. 100 or more times

SCRIPTER: ONLY DISPLAY RESPONSE OPTION 2 IF ECI4 = 2 OR 3 OR 4 OR 5 OR 6

ONLY DISPLAY RESPONSE OPTION 3 IF ECI4 = 3 OR 4 OR 5 OR 6

ONLY DISPLAY RESPONSE OPTION 4 IF ECI4 = 4 OR 5 OR 6

ONLY DISPLAY RESPONSE OPTION 5 IF ECI4 = 5 OR 6

ONLY DISPLAY RESPONSE OPTION 6 IF ECI4 = 6

Base: BID290 = 1

BID290C [S]

[INSERT IMG_290]

Do you now use the **BIDI® Stick Solar (**previously known as **BIDI® Stick Berry Blast)…**

1. Every day

2. Some days

3. Not at all

SCRIPTER: IF ECI351 = 2, DO NOT DISPLAY OPTION 1

SCRIPTER: IF ECI351 = 3, AUTOFILL WITH 3 AND CONTINUE

Base: if BID290 = 1

BID290LT [S]

[INSERT IMG_290]

How many times have you used the **BIDI® Stick Solar (**previously known as **BIDI® Stick Berry Blast)** in your entire life?

1. 1 time, even just a few puffs

2. 2 to 10 times

3. 11 to 20 times

4. 21 to 50 times

5. 51 to 99 times

6. 100 or more times

SCRIPTER: ONLY DISPLAY RESPONSE OPTION 2 IF ECI4 = 2 OR 3 OR 4 OR 5 OR 6

ONLY DISPLAY RESPONSE OPTION 3 IF ECI4 = 3 OR 4 OR 5 OR 6

ONLY DISPLAY RESPONSE OPTION 4 IF ECI4 = 4 OR 5 OR 6

ONLY DISPLAY RESPONSE OPTION 5 IF ECI4 = 5 OR 6

ONLY DISPLAY RESPONSE OPTION 6 IF ECI4 = 6

Base: BID291 = 1

BID291C [S]

[INSERT IMG_291]

Do you now use the **BIDI® Stick Dawn (**previously known as **BIDI® Stick Champion Juice)…**

1. Every day

2. Some days

3. Not at all

SCRIPTER: IF ECI351 = 2, DO NOT DISPLAY OPTION 1

SCRIPTER: IF ECI351 = 3, AUTOFILL WITH 3 AND CONTINUE

Base: if BID291 = 1

BID291LT [S]

[INSERT IMG_291]

How many times have you used the **BIDI® Stick Dawn (**previously known as **BIDI® Stick Champion Juice)** in your entire life?

1. 1 time, even just a few puffs

2. 2 to 10 times

3. 11 to 20 times

4. 21 to 50 times

5. 51 to 99 times

6. 100 or more times

SCRIPTER: ONLY DISPLAY RESPONSE OPTION 2 IF ECI4 = 2 OR 3 OR 4 OR 5 OR 6

ONLY DISPLAY RESPONSE OPTION 3 IF ECI4 = 3 OR 4 OR 5 OR 6

ONLY DISPLAY RESPONSE OPTION 4 IF ECI4 = 4 OR 5 OR 6

ONLY DISPLAY RESPONSE OPTION 5 IF ECI4 = 5 OR 6

ONLY DISPLAY RESPONSE OPTION 6 IF ECI4 = 6

**P30DU – BRANDS**

Base: if (ECI5 ≥ 1) AND (ECI8265 = 1)

[M] (Randomize)

Please look carefully at the brand logos below

In the **past 30 days**, have you used any of these brands of e-cigarettes, even once or twice? (**Select all that you have used**)

P30D265. [INSERT IMG_265]

P30D0. I have not used any of these brands of e-cigarettes in the past 30 days.

SCRIPTER: ANCHOR RESPONSE OPTION P30D0 AND MAKE EXCLUSIVE

SCRIPTER: DISPLAY P30D265 IF ECI8265 = 1

**PATTERNS OF USE – BIDI® Stick ARCTIC**

Base: if BID281C = 1 OR 2 AND BID281LT = 6

BID281I [O]

[INSERT IMG_281]

During the **past 30 days,** on how many days did you use the **BIDI® Stick Arctic (**previously known as **BIDI® Stick Mint Freeze)**?

1. [SCRIPTER INSERT TEXT BOX] days

Min: 0

Max: Response entered in ECI5

CONTROL: NUMERICAL ENTRY ≤ ECI5
SCRIPTER: INSERT NUMERICAL ENTRY BOX TO RESPONSE OPTION 1, NO DECIMALS

SCRIPTER: IF BID281C = 1, DO NOT ACCEPT 0

Base: if BID281C = 1 OR 2 AND BID281LT = 6

BID281D [O]

[INSERT IMG_281]

During the **past 30 days,** about how many **BIDI® Stick Arctic (**previously known as **BIDI® Stick Mint Freeze)** e-cigarettes did you use?

If you haven’t used a full **BIDI® Stick Arctic** (previously known as **BIDI® Stick Mint Freeze**) in the past 30 days, type “0”.

1. [SCRIPTER INSERT TEXT BOX] BIDI® Stick Arctic (Previously known as BIDI® Stick Mint Freeze) e-cigarettes

Min: 0

Max: 60

CONTROL: Max numerical entry 60
SCRIPTER: INSERT NUMERICAL ENTRY BOX TO RESPONSE OPTION 1, NO DECIMALS

SCRIPTER: IF BID281I = 0 AUTOFILL RESPONSE OPTION 1 WITH 99 AND CONTINUE

Base: if BID281C = 1 OR 2 AND BID281LT = 6

BID281Q [S]

[INSERT IMG_281]

Are you seriously thinking about quitting the **BIDI® Stick Arctic (**previously known as **BIDI® Stick Mint Freeze)**? (**Please choose the first answer that fits**)

1. Yes, during the next 30 days

2. Yes, during the next 6 months

3. Yes, during the next 12 months

4. Yes, but not during the next 12 months

5. No, I am not thinking about quitting the BIDI® Stick Arctic (Previously known as BIDI® Stick Mint Freeze)

**PATTERNS OF USE – BIDI® Stick CLASSIC**

Base: if BID282C = 1 OR 2 AND BID282LT = 6

BID282I [O]

[INSERT IMG_282]

During the **past 30 days,** on how many days did you use the **BIDI® Stick Classic (**previously known as **BIDI® Stick Classic Tobacco)**?

1. [SCRIPTER INSERT TEXT BOX] days

Min:0

Max: Response entered in ECI5

CONTROL: NUMERICAL ENTRY ≤ ECI5
SCRIPTER: INSERT NUMERICAL ENTRY BOX TO RESPONSE OPTION 1, NO DECIMALS

SCRIPTER: IF BID282C = 1, DO NOT ACCEPT 0

Base: if BID282C = 1 OR 2 AND BID282LT = 6

BID282D [O]

[INSERT IMG_282]

During the **past 30 days,** about how many **BIDI® Stick Classic (**previously known as **BIDI® Stick Classic Tobacco)** e-cigarettes did you use?

If you haven’t used a full **BIDI® Stick Classic** (previously known as **BIDI® Stick Classic Tobacco**) in the past 30 days, type “0”.

1. [SCRIPTER INSERT TEXT BOX] BIDI® Stick Classic (Previously known as BIDI® Stick Classic Tobacco) e-cigarettes

Min: 0

Max: 60

CONTROL: Max numerical entry 60
SCRIPTER: INSERT NUMERICAL ENTRY BOX TO RESPONSE OPTION 1, NO DECIMALS

SCRIPTER: IF BID282I = 0 AUTOFILL RESPONSE OPTION 1 WITH 99 AND CONTINUE

Base: if BID282C = 1 OR 2 AND BID282LT = 6

BID282Q [S]

[INSERT IMG_282]

Are you seriously thinking about quitting the **BIDI® Stick Classic (**previously known as **BIDI® Stick Classic Tobacco)**? (**Please choose the first answer that fits**)

1. Yes, during the next 30 days

2. Yes, during the next 6 months

3. Yes, during the next 12 months

4. Yes, but not during the next 12 months

5. No, I am not thinking about quitting the BIDI® Stick Classic (Previously known as BIDI® Stick Classic Tobacco)

**PATTERNS OF USE – BIDI® Stick ZEST**

Base: if BID283C = 1 OR 2 AND BID283LT = 6

BID283I [O]

[INSERT IMG_283]

During the **past 30 days,** on how many days did you use the **BIDI® Stick Zest (**previously known as **BIDI® Stick Jungle Juice)**?

1. [SCRIPTER INSERT TEXT BOX] days

Min:0

Max: Response entered in ECI5

CONTROL: NUMERICAL ENTRY ≤ ECI5
SCRIPTER: INSERT NUMERICAL ENTRY BOX TO RESPONSE OPTION 1, NO DECIMALS

SCRIPTER: IF BID283C = 1, DO NOT ACCEPT 0

Base: if BID283C = 1 OR 2 AND BID283LT = 6

BID283D [O]

[INSERT IMG_283]

During the **past 30 days,** about how many **BIDI® Stick Zest (**previously known as **BIDI® Stick Jungle Juice)** e-cigarettes did you use?

If you haven’t used a full **BIDI® Stick Zest** (previously known as **BIDI® Stick Jungle Juice**) in the past 30 days, type “0”.

1. [SCRIPTER INSERT TEXT BOX] BIDI® Stick Zest (Previously known as BIDI® Stick Jungle Juice) e-cigarettes

Min: 0

Max: 60

CONTROL: Max numerical entry 60
SCRIPTER: INSERT NUMERICAL ENTRY BOX TO RESPONSE OPTION 1, NO DECIMALS

SCRIPTER: IF BID283I = 0 AUTOFILL RESPONSE OPTION 1 WITH 99 AND CONTINUE

Base: if BID283C = 1 OR 2 AND BID283LT = 6

BID283Q [S]

[INSERT IMG_283]

Are you seriously thinking about quitting the **BIDI® Stick Zest (**previously known as **BIDI® Stick Jungle Juice)**? (**Please choose the first answer that fits**)

1. Yes, during the next 30 days

2. Yes, during the next 6 months

3. Yes, during the next 12 months

4. Yes, but not during the next 12 months

5. No, I am not thinking about quitting the BIDI® Stick Zest (Previously known as BIDI® Stick Jungle Juice)

**PATTERNS OF USE – BIDI® Stick WINTER**

Base: if BID284C = 1 OR 2 AND BID284LT = 6

BID284I [O]

[INSERT IMG_284]

During the **past 30 days,** on how many days did you use the **BIDI® Stick Winter (**previously known as **BIDI® Stick Lush Ice)**?

1. [SCRIPTER INSERT TEXT BOX] days

Min:0

Max: Response entered in ECI5

CONTROL: NUMERICAL ENTRY ≤ ECI5
SCRIPTER: INSERT NUMERICAL ENTRY BOX TO RESPONSE OPTION 1, NO DECIMALS

SCRIPTER: IF BID284C = 1, DO NOT ACCEPT 0

Base: if BID284C = 1 OR 2 AND BID284LT = 6

BID284D [O]

[INSERT IMG_284]

During the **past 30 days,** about how many **BIDI® Stick Winter (**previously known as **BIDI® Stick Lush Ice)** e-cigarettes did you use?

If you haven’t used a full **BIDI® Stick Winter** (previously known as **BIDI® Stick Lush Ice**) in the past 30 days, type “0”.

1. [SCRIPTER INSERT TEXT BOX] BIDI® Stick Winter (Previously known as BIDI® Stick Lush Ice) e-cigarettes

Min: 0

Max: 60

CONTROL: Max numerical entry 60
SCRIPTER: INSERT NUMERICAL ENTRY BOX TO RESPONSE OPTION 1, NO DECIMALS

SCRIPTER: IF BID284I = 0 AUTOFILL RESPONSE OPTION 1 WITH 99 AND CONTINUE

Base: if BID284C = 1 OR 2 AND BID284LT = 6

BID284Q [S]

[INSERT IMG_284]

Are you seriously thinking about quitting the **BIDI® Stick Winter (**previously known as **BIDI® Stick Lush Ice)**? (**Please choose the first answer that fits**)

1. Yes, during the next 30 days

2. Yes, during the next 6 months

3. Yes, during the next 12 months

4. Yes, but not during the next 12 months

5. No, I am not thinking about quitting the BIDI® Stick Winter (Previously known as BIDI® Stick Lush Ice)

**PATTERNS OF USE – BIDI® Stick TROPIC**

Base: if BID285C = 1 OR 2 AND BID285LT = 6

BID285I [O]

[INSERT IMG_285]

During the **past 30 days,** on how many days did you use the **BIDI® Stick Tropic (**previously known as **BIDI® Stick Blazing Vibe)**?

1. [SCRIPTER INSERT TEXT BOX] days

Min:0

Max: Response entered in ECI5

CONTROL: NUMERICAL ENTRY ≤ ECI5
SCRIPTER: INSERT NUMERICAL ENTRY BOX TO RESPONSE OPTION 1, NO DECIMALS

SCRIPTER: IF BID285C = 1, DO NOT ACCEPT 0

Base: if BID285C = 1 OR 2 AND BID285LT = 6

BID285D [O]

[INSERT IMG_285]

During the **past 30 days,** about how many **BIDI® Stick Tropic (**previously known as **BIDI® Stick Blazing Vibe)** e-cigarettes did you use?

If you haven’t used a full **BIDI® Stick Tropic** (previously known as **BIDI® Stick Blazing Vibe**) in the past 30 days, type “0”.

1. [SCRIPTER INSERT TEXT BOX] BIDI® Stick Tropic (Previously known as BIDI® Stick Blazing Vibe) e-cigarettes

Min: 0

Max: 60

CONTROL: Max numerical entry 60
SCRIPTER: INSERT NUMERICAL ENTRY BOX TO RESPONSE OPTION 1, NO DECIMALS

SCRIPTER: IF BID285I = 0 AUTOFILL RESPONSE OPTION 1 WITH 99 AND CONTINUE

Base: if BID285C = 1 OR 2 AND BID285LT = 6

BID285Q [S]

[INSERT IMG_285]

Are you seriously thinking about quitting the **BIDI® Stick Tropic (**previously known as **BIDI® Stick Blazing Vibe)**? (**Please choose the first answer that fits**)

1. Yes, during the next 30 days

2. Yes, during the next 6 months

3. Yes, during the next 12 months

4. Yes, but not during the next 12 months

5. No, I am not thinking about quitting the BIDI® Stick Tropic (Previously known as BIDI® Stick Blazing Vibe)

**PATTERNS OF USE – BIDI® Stick GOLD**

Base: if BID286C = 1 OR 2 AND BID286LT = 6

BID286I [O]

[INSERT IMG_286]

During the **past 30 days,** on how many days did you use the **BIDI® Stick Gold (**previously known as **BIDI® Stick Fruity Mango)**?

1. [SCRIPTER INSERT TEXT BOX] days

Min:0

Max: Response entered in ECI5

CONTROL: NUMERICAL ENTRY ≤ ECI5
SCRIPTER: INSERT NUMERICAL ENTRY BOX TO RESPONSE OPTION 1, NO DECIMALS

SCRIPTER: IF BID286C = 1, DO NOT ACCEPT 0

Base: if BID286C = 1 OR 2 AND BID286LT = 6

BID286D [O]

INSERT IMG_286]

During the **past 30 days,** about how many **BIDI® Stick Gold (**previously known as **BIDI® Stick Fruity Mango)** e-cigarettes did you use?

If you haven’t used a full **BIDI® Stick Gold** (previously known as **BIDI® Stick Fruity Mango**) in the past 30 days, type “0”.

1. [SCRIPTER INSERT TEXT BOX] BIDI® Stick Gold (Previously known as BIDI® Stick Fruity Mango) e-cigarettes

Min: 0

Max: 60

CONTROL: Max numerical entry 60
SCRIPTER: INSERT NUMERICAL ENTRY BOX TO RESPONSE OPTION 1, NO DECIMALS

SCRIPTER: IF BID286I = 0 AUTOFILL RESPONSE OPTION 1 WITH 99 AND CONTINUE

Base: if BID286C = 1 OR 2 AND BID286LT = 6

BID286Q [S]

[INSERT IMG_286]

Are you seriously thinking about quitting the **BIDI® Stick Gold (**previously known as **BIDI® Stick Fruity Mango)**? (**Please choose the first answer that fits**)

1. Yes, during the next 30 days

2. Yes, during the next 6 months

3. Yes, during the next 12 months

4. Yes, but not during the next 12 months

5. No, I am not thinking about quitting the BIDI® Stick Gold (Previously known as BIDI® Stick Fruity Mango)

**PATTERNS OF USE – BIDI® Stick MARIGOLD**

Base: if BID287C = 1 OR 2 AND BID287LT = 6

BID287I [O]

[INSERT IMG_287]

During the **past 30 days,** on how many days did you use the **BIDI® Stick Marigold (**previously known as **BIDI® Stick Icy Mango)**?

1. [SCRIPTER INSERT TEXT BOX] days

Min:0

Max: Response entered in ECI5

CONTROL: NUMERICAL ENTRY ≤ ECI5
SCRIPTER: INSERT NUMERICAL ENTRY BOX TO RESPONSE OPTION 1, NO DECIMALS

SCRIPTER: IF BID287C = 1, DO NOT ACCEPT 0

Base: if BID287C = 1 OR 2 AND BID287LT = 6

BID287D [O]

[INSERT IMG_287]

During the **past 30 days,** about how many **BIDI® Stick Marigold (**previously known as **BIDI® Stick Icy Mango)** e-cigarettes did you use?

If you haven’t used a full **BIDI® Stick Marigold** (previously known as **BIDI® Stick Icy Mango**) in the past 30 days, type “0”.

1. [SCRIPTER INSERT TEXT BOX] BIDI® Stick Marigold (Previously known as BIDI® Stick Icy Mango) e-cigarettes

Min: 0

Max: 60

CONTROL: Max numerical entry 60
SCRIPTER: INSERT NUMERICAL ENTRY BOX TO RESPONSE OPTION 1, NO DECIMALS

SCRIPTER: IF BID287I = 0 AUTOFILL RESPONSE OPTION 1 WITH 99 AND CONTINUE

Base: if BID287C = 1 OR 2 AND BID287LT = 6

BID287Q [S]

[INSERT IMG_287]

Are you seriously thinking about quitting the **BIDI® Stick Marigold (**previously known as **BIDI® Stick Icy Mango)**? (**Please choose the first answer that fits**)

1. Yes, during the next 30 days

2. Yes, during the next 6 months

3. Yes, during the next 12 months

4. Yes, but not during the next 12 months

5. No, I am not thinking about quitting the BIDI® Stick Marigold (Previously known as BIDI® Stick Icy Mango)

**PATTERNS OF USE – BIDI® Stick REGAL**

Base: if BID288C = 1 OR 2 AND BID288LT = 6

BID288I [O]

[INSERT IMG_288]

During the **past 30 days,** on how many days did you use the **BIDI® Stick Regal (**previously known as **BIDI® Stick Dragon Venom)**?

1. [SCRIPTER INSERT TEXT BOX] days

Min:0

Max: Response entered in ECI5

CONTROL: NUMERICAL ENTRY ≤ ECI5
SCRIPTER: INSERT NUMERICAL ENTRY BOX TO RESPONSE OPTION 1, NO DECIMALS

SCRIPTER: IF BID288C = 1, DO NOT ACCEPT 0

Base: if BID288C = 1 OR 2 AND BID288LT = 6

BID288D [O]

INSERT IMG_288]

During the **past 30 days,** about how many **BIDI® Stick Regal (**previously known as **BIDI® Stick Dragon Venom)** e-cigarettes did you use?

If you haven’t used a full **BIDI® Stick Regal** (previously known as **BIDI® Stick Dragon Venom**) in the past 30 days, type “0”.

1. [SCRIPTER INSERT TEXT BOX] BIDI® Stick Regal (Previously known as BIDI® Stick Dragon Venom) e-cigarettes

Min: 0

Max: 60

CONTROL: Max numerical entry 60
SCRIPTER: INSERT NUMERICAL ENTRY BOX TO RESPONSE OPTION 1, NO DECIMALS

SCRIPTER: IF BID288I = 0 AUTOFILL RESPONSE OPTION 1 WITH 99 AND CONTINUE

Base: if BID288C = 1 OR 2 AND BID288LT = 6

BID288Q [S]

[INSERT IMG_288]

Are you seriously thinking about quitting the **BIDI® Stick Regal (**previously known as **BIDI® Stick Dragon Venom)**? (**Please choose the first answer that fits**)

1. Yes, during the next 30 days

2. Yes, during the next 6 months

3. Yes, during the next 12 months

4. Yes, but not during the next 12 months

5. No, I am not thinking about quitting the BIDI® Stick Regal (Previously known as BIDI® Stick Dragon Venom)

**PATTERNS OF USE – BIDI® Stick SUMMER**

Base: if BID289C = 1 OR 2 AND BID289LT = 6

BID289I [O]

[INSERT IMG_289]

During the **past 30 days,** on how many days did you use the **BIDI® Stick Summer (**previously known as **BIDI® Stick Kick Start)**?

1. [SCRIPTER INSERT TEXT BOX] days

Min:0

Max: Response entered in ECI5

CONTROL: NUMERICAL ENTRY ≤ ECI5
SCRIPTER: INSERT NUMERICAL ENTRY BOX TO RESPONSE OPTION 1, NO DECIMALS

SCRIPTER: IF BID289C = 1, DO NOT ACCEPT 0

Base: if BID289C = 1 OR 2 AND BID289LT = 6

BID289D [O]

[INSERT IMG_289]

During the **past 30 days,** about how many **BIDI® Stick Summer (**previously known as **BIDI® Stick Kick Start)** e-cigarettes did you use?

If you haven’t used a full **BIDI® Stick Summer** (previously known as **BIDI® Stick Kick Start**) in the past 30 days, type “0”.

1. [SCRIPTER INSERT TEXT BOX] BIDI® Stick Summer (Previously known as BIDI® Stick Kick Start) e-cigarettes

Min: 0

Max: 60

CONTROL: Max numerical entry 60
SCRIPTER: INSERT NUMERICAL ENTRY BOX TO RESPONSE OPTION 1, NO DECIMALS

SCRIPTER: IF BID289I = 0 AUTOFILL RESPONSE OPTION 1 WITH 99 AND CONTINUE

Base: if BID289C = 1 OR 2 AND BID289LT = 6

BID289Q [S]

[INSERT IMG_289]

Are you seriously thinking about quitting the **BIDI® Stick Summer (**previously known as **BIDI® Stick Kick Start)**? (**Please choose the first answer that fits**)

1. Yes, during the next 30 days

2. Yes, during the next 6 months

3. Yes, during the next 12 months

4. Yes, but not during the next 12 months

5. No, I am not thinking about quitting the BIDI® Stick Summer (Previously known as BIDI® Stick Kick Start)

**PATTERNS OF USE – BIDI® Stick SOLAR**

Base: if BID290C = 1 OR 2 AND BID290LT = 6

BID290I [O]

[INSERT IMG_290]

During the **past 30 days,** on how many days did you use the **BIDI® Stick Solar (**previously known as **BIDI® Stick Berry Blast)**?

1. [SCRIPTER INSERT TEXT BOX] days

Min:0

Max: Response entered in ECI5

CONTROL: NUMERICAL ENTRY ≤ ECI5
SCRIPTER: INSERT NUMERICAL ENTRY BOX TO RESPONSE OPTION 1, NO DECIMALS

SCRIPTER: IF BID290C = 1, DO NOT ACCEPT 0

Base: if BID290C = 1 OR 2 AND BID290LT = 6

BID290D [O]

[INSERT IMG_290]

During the **past 30 days,** about how many **BIDI® Stick Solar (**previously known as **BIDI® Stick Berry Blast)** e-cigarettes did you use?

If you haven’t used a full **BIDI® Stick Solar** (previously known as **BIDI® Stick Berry Blast**) in the past 30 days, type “0”.

1. [SCRIPTER INSERT TEXT BOX] BIDI® Stick Solar (Previously known as BIDI® Stick Berry Blast) e-cigarettes

Min: 0

Max: 60

CONTROL: Max numerical entry 60
SCRIPTER: INSERT NUMERICAL ENTRY BOX TO RESPONSE OPTION 1, NO DECIMALS

SCRIPTER: IF BID290I = 0 AUTOFILL RESPONSE OPTION 1 WITH 99 AND CONTINUE

Base: if BID290C = 1 OR 2 AND BID290LT = 6

BID290Q [S]

[INSERT IMG_290]

Are you seriously thinking about quitting the **BIDI® Stick Solar (**previously known as **BIDI® Stick Berry Blast)**? (**Please choose the first answer that fits**)

1. Yes, during the next 30 days

2. Yes, during the next 6 months

3. Yes, during the next 12 months

4. Yes, but not during the next 12 months

5. No, I am not thinking about quitting the BIDI® Stick Solar (Previously known as BIDI® Stick Berry Blast)

**PATTERNS OF USE – BIDI® Stick DAWN**

Base: if BID291C = 1 OR 2 AND BID291LT = 6

BID291I [O]

[INSERT IMG_291]

During the **past 30 days,** on how many days did you use the **BIDI® Stick Dawn (**previously known as **BIDI® Stick Champion Juice)**?

1. [SCRIPTER INSERT TEXT BOX] days

Min:0

Max: Response entered in ECI5

CONTROL: NUMERICAL ENTRY ≤ ECI5
SCRIPTER: INSERT NUMERICAL ENTRY BOX TO RESPONSE OPTION 1, NO DECIMALS

SCRIPTER: IF BID291C = 1, DO NOT ACCEPT 0

Base: if BID291C = 1 OR 2 AND BID291LT = 6

BID291D [O]

[INSERT IMG_291]

During the **past 30 days,** about how many **BIDI® Stick Dawn (**previously known as **BIDI® Stick Champion Juice)** e-cigarettes did you use?

If you haven’t used a full **BIDI® Stick Dawn** (previously known as **BIDI® Stick Champion Juice**) in the past 30 days, type “0”.

1. [SCRIPTER INSERT TEXT BOX] BIDI® Stick Dawn (Previously known as BIDI® Stick Champion Juice) e-cigarettes

Min: 0

Max: 60

CONTROL: Max numerical entry 60
SCRIPTER: INSERT NUMERICAL ENTRY BOX TO RESPONSE OPTION 1, NO DECIMALS

SCRIPTER: IF BID291I = 0 AUTOFILL RESPONSE OPTION 1 WITH 99 AND CONTINUE

Base: if BID291C = 1 OR 2 AND BID291LT = 6

BID291Q [S]

[INSERT IMG_291]

Are you seriously thinking about quitting the **BIDI® Stick Dawn (**previously known as **BIDI® Stick Champion Juice)**? (**Please choose the first answer that fits**)

1. Yes, during the next 30 days

2. Yes, during the next 6 months

3. Yes, during the next 12 months

4. Yes, but not during the next 12 months

5. No, I am not thinking about quitting the BIDI® Stick Dawn (Previously known as BIDI® Stick Champion Juice)

Base: All respondents

END_INT

Thank you for taking part in our survey, we appreciate your time.
