# Supplementary material for: When it comes to assessing the impact of e-cigarettes, estimates of device prevalence matter: the BIDI Stick disposable device
Source: Harm Reduct J. 2023 Jul 5;20:85. doi: 10.1186/s12954-023-00820-y (PMC10324178; doi:10.1186/s12954-023-00820-y)
Supplement: Supplementary file 2 — Additional file 2: Youth survey instrument. [file 12954_2023_820_MOESM2_ESM.docx]

**I. ROSTERING EXERCISE**

Base: all respondents

R01 [Q]

First, how many children between the ages of 13 and 17 live in your household at least 50% of the time?

If you do not have any children between the ages 13 and 17 who live in your household at least 50% of the time, please enter zero.

*Scripter, prompt numeric box range 0-15; do not allow decimals.*

____ [range 0 - 15]

*Scripter, terminate if refused; if R1=0, insert standard close and terminate.*

Base: if R01 = 1

R02 [Q]

And are you the parent of this child between the ages of 13 and 17?

1. Yes

2. No

*Scripter, terminate if refused; if R02=2, insert standard close and terminate.*

If R02=1

R03 [Q]

Now we would like to ask you a little more about the child between the ages of 13 and 17 in your household for whom you are the parent. How old is your child?

*Scripter, prompt numeric box range 13 - 17; do not allow decimals*

____ [range 13 – 17]

*Scripter, terminate if refused; if R03≠13-17, insert standard close and terminate.*

Base: if R02 = 1

R04 [S]

Is this child male or female?

1. Male

2. Female

Base: if R01 > 1

R05a [S] PROMPT up to 2 times for answers to Age and Gender

*Scripter, if R02 is = 1, skip to S1*

We would like to determine which of the children 13-17 living in your household should complete the rest of the survey.

To allow us to do this, we need a little bit more information. First, for each child aged 13 to 17 in your household, enter their age and indicate if they are male or female. SCRIPTER INSERT NUMBER OF ROWS EQUAL TO NUMBER OF CHILDREN IN R01

*Household members in row:*

1. Child 1

2. Child 2

3. Child 3

4. Child 4

5. Child 5

6. Child 6

7. Child 7

8. Child 8

9. Child 9

10. Child 10

*Answers in second column:*

1. Male

2. Female

*SCRIPTER: Show grid with same number of lines as R01 response. First column: heading “Age” min.=13, max.=17. Second column: heading “Gender”, show radio buttons.*

Base: if R01 > 1

R05b [S]

PROMPT up to 2 times for answers

*Scripter, if R02 is = 1, skip to S1*

For each child 13-17 living in your household, tell us if you are the parent.

Select an answer from each row in the grid.

Household members in row:

1. Child 1

2. Child 2

3. Child 3

4. Child 4

5. Child 5

6. Child 6

7. Child 7

8. Child 8

9. Child 9

10. Child 10

Answers in column:

1. Parent

2. Not a parent

*SCRIPTER: Show grid with same number of lines as household members under age 18 in HHCOMP1. Add Age and Gender in child list (e.g., Child 3 – Male, 17). Column heading “Relationship”, show radio buttons.*

*Scripter, terminate if all children = refused or 2; insert standard close and terminate.*

*Scripter, if more than one child is entered above, please randomly select one child ages 13 and 17 to take the survey and show R08. If all table entries are not aged between 13 and 17 years old, terminate and insert standard close. Record in DOV_CHILD. TERMINATE IF NONE OF THE CHILDREN IS BETWEEN 13 – 17.*

*IF A CHILD IS ENTERED WHO IS NOT 13-17 THAT CHILD CANNOT BE SELECTED TO TAKE THE SURVEY.*

*Scripter, **create DOVs for child age and gender ***

| **DOV_Age** |  |
| --- | --- |
| IF R02=1 | DOV_Age=R03 |
| IF R01 >1 | DOV_Age= Age of child selected in R05 |
| **DOV_Gender** |  |
| IF R02=1 | DOV_Gender=R04 |
| IF R01 >1 | DOV_Gender= Gender of child selected in R05 |
|  |  |
|  |  |
|  |  |

**II. PARENTAL CONSENT & SCREENER**

S1 [S] [Prompt up to 2 times for an answer]

IF R02=1 INSERT: Your child has been selected to complete the survey.

IF R01>1 INSERT: Your [INSERT DOV_AGE year old DOV_GENDER FROM R05] child has been selected to complete the survey.

*Scripter: if respondent refuses terminate.*

First, let’s complete your portion of the survey.

Base: if S1=YES

R06 [S]

What grade is your [R01>1 INSERT: DOV_AGE year old DOV_GENDER FROM R05] child in at school?

1. 6th grade

2. 7th grade

3. 8th grade

4. 9th grade

5. 10th grade

6. 11th grade

7. 12th grade

8. Ungraded or other grade

| DOV_Grade | DOV_Grade = R06 |
| --- | --- |

Base: S1=yes

R08 [S] [PROMPT]

This next question is about Hispanic ethnicity. Is your [R01>1 INSERT: DOV_AGE year old DOV_GENDER FROM R05] child of Spanish, Hispanic, or Latino descent?

1. No, he/she is not

2. Yes, Mexican, Mexican-American, Chicano

3. Yes, Puerto Rican

4. Yes, Cuban, Cuban American

5. Yes, other Spanish, Hispanic, or Latino group (Please specify, for example Argentinean, Colombian, Dominican, Nicaraguan, Salvadoran, Spaniard, and s

o on) [O]

Base: S1=yes

R09 [M] [PROMPT]

Please tell us what you consider your [R01>1 INSERT: DOV_AGE year old DOV_GENDER FROM R05] child’s racial background to be. We greatly appreciate your effort to describe the child’s racial background using the standard categories provided. These race categories may not fully describe the child, but they do match those used by the Census Bureau.

Please check one or more categories below to indicate what race(s) you consider the child to be.

1. White

2. Black or African American

3. American Indian or Alaska Native

4. Asian

5. Native Hawaiian or other Pacific Islander

6. Some other race [O]

*SCRIPTER: Create Data-only variable TPPETHM by using the below logic involving responses to R08 and R09*

**Variable name:** TPPETHM [S]

**Variable Text:**  Census Ethnicity demographic

**Response list:**

1.  White, Non-Hispanic

2.  Black, Non-Hispanic

3.  Other, Non-Hispanic

4.  Hispanic

5.  2+ Races, Non-Hispanic

Count numraces=R09 1 R09 2 R09 3 R09 4 R09 5 R09 6 (1).

| **R08** | **R09** | **TPPETHM** |
| --- | --- | --- |
| R08=1 | R09=1 | 1 |
| R08=1 | R09=2 | 2 |
| R08=1 | R09=3 or R09=4 or R09=5 or R09=6 | 3 |
| R08=1 | numraces > 1 | 5 |
| R08=2 OR 3 OR 4 OR 8 | (numraces=1 or numraces>1) | 4 |
| REFUSED | Any value | MISSING |
| Any value | REFUSED | MISSING |
| R08=2 OR 3 OR 4 OR 8 | REFUSED | 4 |

Base: S1=Yes

PROMPT ONCE

R10. Is your [R01>1 INSERT: DOV_AGE year old DOV_GENDER FROM R05] child currently available to take the survey?

1. Yes

2. No, but available at a later time

*Scripter: If refused terminate.*

Base: if answer is 1, Yes

*Scripter, please display*

Please have your [R01>1 INSERT: DOV_AGE year old DOV_GENDER FROM R05] child come to the computer right now to take the survey.

Base: if answer is 2, No

*Scripter, please display*

Please have your [R01>1 INSERT: DOV_AGE year old DOV_GENDER FROM R05] child take the survey as soon as possible. They can access the survey through your Member Page. Please remind your [R01>1 INSERT: DOV_AGE year old DOV_GENDER FROM R05] child to take only this survey and not to complete any other surveys.

*Scripter: Suspend survey, but allow respondents to re-enter it*

**IV. YOUTH CONSENT**

Base: S1 = Yes

[DISPLAY TEXT] [prompt up to 2 times]

**QUESTIONNAIRE**

**1. COMBUSTIBLE CIGARETTES**

Base: all respondents

CIGINT [Descriptor Text]

The first questions are about smoking cigarettes (ones that have to be lit and burned).

Base: all respondents

CIG1 [S]

Have you ever tried cigarette smoking, even one or two puffs?

1. Yes

2. No

SCRIPTER: REQUEST RESPONSE

Base: if CIG1 = 1

CIG2 [S]

How old were you when you **first tried** cigarette smoking, even one or two puffs?

1.[SCRIPTER INSERT NUMERICAL ENTRY BOX] years old

CONTROL: ENTERED RESPONSE ≤ Age

SCRIPTER: INSERT NUMERICAL ENTRY BOX INTO RESPONSE OPTION 1, NO DECIMALS

Base: if CIG1 = 1

CIG3 [S]

How many cigarettes have you smoked in your **entire life**? A pack usually has 20 cigarettes in it.

1. 1 or more puffs but never a whole cigarette

2. 1 cigarette

3. 2 to 5 cigarettes

4. 6 to 15 cigarettes (about 1/2 a pack total)

5. 16 to 25 cigarettes (about 1 pack total)

6. 26 to 99 cigarettes (more than 1 pack, but less than 5 packs)

7. 100 or more cigarettes (5 or more packs)

Base: if CIG1 = 1

CIG4 [S]

In the **past 30 days**, on how many days did you smoke cigarettes?

1. [ENTER NUMBER] Day(s)

CONTROL:

Min: 0

Max: 30

SCRIPTER: INSERT NUMERICAL ENTRY BOX INTO RESPONSE OPTION 1, NO DECIMALS

SCRIPTER: REQUEST RESPONSE

Base: if CIG1 = 1

CIG5 [S]

When was the **last time** you smoked a cigarette, even one or two puffs?

1. Earlier today

2. Not today but sometime during the past 7 days

3. Not during the past 7 days but sometime during the past 30 days

4. Not during the past 30 days but sometime during the past 6 months

5. Not during the past 6 months but sometime during the past year

6. 1 to 4 years ago

7. 5 or more years ago

SCRIPTER: DO NOT DISPLAY RESPONSE OPTIONS 4 AND 5 AND 6 AND 7 if CIG4 ≥ 1

DO NOT DISPLAY RESPONSE OPTIONS 1 AND 2 AND 3 if CIG4 = 0

Base: CIG4 ≥ 1

CIG6 [S]

In the past 30 days, **on the days you smoked**, how many cigarettes did you smoke per day? A pack usually has 20 cigarettes in it.

1. Less than 1 cigarette per day

2. 1 cigarette per day

3. 2 to 5 cigarettes per day

4. 6 to 10 cigarettes per day

5. 11 to 20 cigarettes per day

6. More than 20 cigarettes per day

Base: CIG4 ≥ 1

CIG7 [S]

Are you seriously thinking about quitting **cigarettes**? (Please choose the first answer that fits)

1. Yes, during the next 30 days

2. Yes, during the next 6 months

3. Yes, during the next 12 months

4. Yes, but not during the next 12 months

5. No, I am not thinking about quitting cigarettes

Base: if CIG1 = 2

CIG8 [S]

Have you ever been curious about smoking a cigarette?

1. Very curious

2. Somewhat curious

3. A little curious

4. Not at all curious

998. DON’T KNOW

Base: if CIG1 = 2

CIG9 [S]

Do you think you will smoke a cigarette in the next year?

1. Definitely yes

2. Probably yes

3. Probably not

4. Definitely not

998. DON’T KNOW

Base: if CIG1 = 2

CIG10 [S]

Do you think that you will try a cigarette soon?

1. Definitely yes

2. Probably yes

3. Probably not

4. Definitely not

998. DON’T KNOW

Base: if CIG1 = 2

CIG11 [S]

If one of your best friends were to offer you a cigarette, would you smoke it?

1. Definitely yes

2. Probably yes

3. Probably not

4. Definitely not

998. DON’T KNOW

**2. GENERIC E-CIGARETTES**

Base: all respondents

ECIG_INT [Descriptor text]

The next several questions are about electronic cigarettes or e-cigarettes, such as JUUL, Vuse, blu, and Logic.. E-cigarettes are battery powered devices that usually contain a nicotine-based liquid that is vaporized and inhaled. You may also know them as e-cigs, vape-pens, e-hookahs, vapes, or mods.

Base: all respondents

ECI1 [S]

Have you ever seen or heard of e-cigarettes before this study?

1. Yes

2. No

SCRIPTER: REQUEST RESPONSE

Base: ECI1 = 1

ECI2 [S]

Have you **ever used** an e-cigarette, even one or two times?

1. Yes

2. No

SCRIPTER: REQUEST RESPONSE

Base: if ECI2 = 1

ECI3 [S]

How old were you when you **first used** an e-cigarette, even once or twice?

1. [SCRIPTER INSERT NUMERICAL ENTRY BOX] years old

CONTROL: ENTERED RESPONSE ≤ Age

SCRIPTER: INSERT NUMERICAL ENTRY BOX TO RESPONSE OPTION 1, NO DECIMALS

Base: if ECI2 = 1

ECI4 [S]

How many times have you used an e-cigarette in your **entire life**?

1. 1 time, even just a few puffs

2. 2 to 10 times

3. 11 to 20 times

4. 21 to 50 times

5. 51 to 99 times

6. 100 or more times

Base: if ECI2 = 1

ECI5 [S]

During the **past 30 days**, on how many days did you use an e-cigarette?

1. [ENTER NUMBER] Day(s)

Min: 0

Max: 30

SCRIPTER: INSERT NUMERICAL ENTRY BOX TO RESPONSE OPTION 1, NO DECIMALS

SCRIPTER: REQEUST RESPONSE

Base: if ECI2 = 1

ECI6 [S]

When was the **last time** you used an e-cigarette, even one or two puffs? (Please choose the first answer that fits)

1. Earlier today

2. Not today but sometime during the past 7 days

3. Not during the past 7 days but sometime during the past 30 days

4. Not during the past 30 days but sometime during the past 6 months

5. Not during the past 6 months but sometime during the past year

6. 1 to 4 years ago

7. 5 or more years ago

SCRIPTER: DO NOT DISPLAY RESPONSE OPTIONS 4 AND 5 AND 6 AND 7 IF ECI5 ≥ 1

SCRIPTER: DO NOT DISPLAY RESPONSE OPTIONS 1 AND 2 AND 3 IF ECI5 = 0

SCRIPTER: DISPLAY OPTIONS 1 TO 7 IF ECI5 = -1

Base: if ECI5 ≥ 1

[M]

Which flavors of e-liquid have you used in an e-cigarette in **the past 30 days**? Choose all that apply

ECIF1. Tobacco

ECIF2. Menthol/Mint

ECIF3. Fruit

ECIF4. Dessert (such as Muffin, Ice cream, custard or other desserts)

ECIF5. Alcoholic drink (such as bourbon, piña colada, mojito or other alcoholic drinks)

ECIF6. Other beverages (such as cola, energy drink, milk or other non-alcoholic beverages)

ECIF7. Candy (such as bubble gum, cotton candy, gummy bears or other candy)

ECIF8. Other sweets (such as chocolate, caramel, vanilla or other sweets)

ECIF9. Coffee/Tea (such as caffè latte, cappuccino, tea or other)
ECIF10. Spices (such as cinnamon, clove, licorice or other spices)

ECIF11. Nuts (such as hazelnut, peanut (butter), pecan or other nuts)
ECIF12. Another flavor not listed here [Specify: SCRIPTER INSERT TEXT BOX]

ECIF998. I DON’T KNOW

SCRIPTER: RESPONSE OPTION ECIF998 IS EXCLUSIVE

Base: if ECI2 = 1

[M] (Randomize)

Please look carefully at the brand logos below

Have you ever used any of these brands of e-cigarettes, even once or twice? (**Select all that you have ever used**)

ECI8265. [INSERT IMG_265]

ECI80. I have not used any of these brands of e-cigarettes

SCRIPTER: ANCHOR RESPONSE OPTION ECI80 AND MAKE EXCLUSIVE

Base: if ECI2 = 2

ECI11 [S]

Have you ever been curious about using an e-cigarette?

1. Very curious

2. Somewhat curious

3. A little curious

4. Not at all curious

998. DON’T KNOW

Base: if ECI2 = 2

ECI12 [S]

Do you think you will use an e-cigarette in the next year?

1. Definitely yes

2. Probably yes

3. Probably not

4. Definitely not

998. DON’T KNOW

Base: if ECI2 = 2

ECI13 [S]

Do you think that you will try an e-cigarette soon?

1. Definitely yes

2. Probably yes

3. Probably not

4. Definitely not

998. DON’T KNOW

Base: if ECI2 = 2

ECI14 [S]

If one of your best friends were to offer you an e-cigarette, would you use it?

1. Definitely yes

2. Probably yes

3. Probably not

4. Definitely not

998. DON’T KNOW

Base: ECI5 ≥ 1

ECI15 [S]

Are you seriously thinking about quitting **e-cigarettes**?

1. Yes, during the next 30 days

2. Yes, during the next 6 months

3. Yes, during the next 12 months

4. Yes, but not during the next 12 months

5. No, I am not seriously thinking about quitting

Base: IF ECI8265 = 1

ECIINT2 [Descriptor text]

The next few questions will ask you about your use of specific e-cigarette devices.

In answering these questions, please ignore the device color. Please focus only on the device itself.

**EVER USE – BIDI® Stick**

Base: if ECI8265 = 1

[M] (Randomize)

You said that you have used a **BIDI® Stick** e-cigarette.

Below are the pictures of different e-cigarettes that are made by **BIDI® Stick**,

Which of these **BIDI® Stick** e-cigarettes have you ever used, even once or twice? (Check all that apply)

BID281. [INSERT IMG_281]

BID282. [INSERT IMG_282]

BID283. [INSERT IMG_283]

BID284. [INSERT IMG_284]

BID285. [INSERT IMG_285]

BID286. [INSERT IMG_286]

BID287. [INSERT IMG_287]

BID288. [INSERT IMG_288]

BID289. [INSERT IMG_289]

BID290. [INSERT IMG_290]

BID291. [INSERT IMG_291]

BID0. I have not used any of these e-cigarettes

SCRIPTER: ANCHOR RESPONSE OPTION BID0 AND MAKE EXCLUSIVE

Base: if BID281 = 1

BID281LT [S]

[INSERT IMG_281]

How many times have you used the **BIDI® Stick Arctic (**previously known as **BIDI® Stick** **Mint Freeze)** in your entire life?

1. 1 time, even just a few puffs

2. 2 to 10 times

3. 11 to 20 times

4. 21 to 50 times

5. 51 to 99 times

6. 100 or more times

SCRIPTER: ONLY DISPLAY RESPONSE OPTION 2 IF ECI4 = 2 OR 3 OR 4 OR 5 OR 6

ONLY DISPLAY RESPONSE OPTION 3 IF ECI4 = 3 OR 4 OR 5 OR 6

ONLY DISPLAY RESPONSE OPTION 4 IF ECI4 = 4 OR 5 OR 6

ONLY DISPLAY RESPONSE OPTION 5 IF ECI4 = 5 OR 6

ONLY DISPLAY RESPONSE OPTION 6 IF ECI4 = 6

Base: if BID282 = 1

BID282LT [S]

[INSERT IMG_282]

How many times have you used the **BIDI® Stick Classic (**previously known as **BIDI® Stick** **Classic Tobacco)** in your entire life?

1. 1 time, even just a few puffs

2. 2 to 10 times

3. 11 to 20 times

4. 21 to 50 times

5. 51 to 99 times

6. 100 or more times

SCRIPTER: ONLY DISPLAY RESPONSE OPTION 2 IF ECI4 = 2 OR 3 OR 4 OR 5 OR 6

ONLY DISPLAY RESPONSE OPTION 3 IF ECI4 = 3 OR 4 OR 5 OR 6

ONLY DISPLAY RESPONSE OPTION 4 IF ECI4 = 4 OR 5 OR 6

ONLY DISPLAY RESPONSE OPTION 5 IF ECI4 = 5 OR 6

ONLY DISPLAY RESPONSE OPTION 6 IF ECI4 = 6

Base: if BID283 = 1

BID283LT [S]

[INSERT IMG_283]

How many times have you used the **BIDI® Stick Zest (**previously known as **BIDI® Stick** **Jungle Juice)** in your entire life?

1. 1 time, even just a few puffs

2. 2 to 10 times

3. 11 to 20 times

4. 21 to 50 times

5. 51 to 99 times

6. 100 or more times

SCRIPTER: ONLY DISPLAY RESPONSE OPTION 2 IF ECI4 = 2 OR 3 OR 4 OR 5 OR 6

ONLY DISPLAY RESPONSE OPTION 3 IF ECI4 = 3 OR 4 OR 5 OR 6

ONLY DISPLAY RESPONSE OPTION 4 IF ECI4 = 4 OR 5 OR 6

ONLY DISPLAY RESPONSE OPTION 5 IF ECI4 = 5 OR 6

ONLY DISPLAY RESPONSE OPTION 6 IF ECI4 = 6

Base: if BID284 = 1

BID284LT [S]

[INSERT IMG_284]

How many times have you used the **BIDI® Stick Winter (**previously known as **BIDI® Stick** **Lush Ice)** in your entire life?

1. 1 time, even just a few puffs

2. 2 to 10 times

3. 11 to 20 times

4. 21 to 50 times

5. 51 to 99 times

6. 100 or more times

SCRIPTER: ONLY DISPLAY RESPONSE OPTION 2 IF ECI4 = 2 OR 3 OR 4 OR 5 OR 6

ONLY DISPLAY RESPONSE OPTION 3 IF ECI4 = 3 OR 4 OR 5 OR 6

ONLY DISPLAY RESPONSE OPTION 4 IF ECI4 = 4 OR 5 OR 6

ONLY DISPLAY RESPONSE OPTION 5 IF ECI4 = 5 OR 6

ONLY DISPLAY RESPONSE OPTION 6 IF ECI4 = 6

Base: if BID285 = 1

BID285LT [S]

[INSERT IMG_285]

How many times have you used the **BIDI® Stick Tropic (**previously known as **BIDI® Stick** **Blazing Vibe)** in your entire life?

1. 1 time, even just a few puffs

2. 2 to 10 times

3. 11 to 20 times

4. 21 to 50 times

5. 51 to 99 times

6. 100 or more times

SCRIPTER: ONLY DISPLAY RESPONSE OPTION 2 IF ECI4 = 2 OR 3 OR 4 OR 5 OR 6

ONLY DISPLAY RESPONSE OPTION 3 IF ECI4 = 3 OR 4 OR 5 OR 6

ONLY DISPLAY RESPONSE OPTION 4 IF ECI4 = 4 OR 5 OR 6

ONLY DISPLAY RESPONSE OPTION 5 IF ECI4 = 5 OR 6

ONLY DISPLAY RESPONSE OPTION 6 IF ECI4 = 6

Base: if BID286 = 1

BID286LT [S]

[INSERT IMG_286]

How many times have you used the **BIDI® Stick Gold (**previously known as **BIDI® Stick** **Fruity Mango)** in your entire life?

1. 1 time, even just a few puffs

2. 2 to 10 times

3. 11 to 20 times

4. 21 to 50 times

5. 51 to 99 times

6. 100 or more times

SCRIPTER: ONLY DISPLAY RESPONSE OPTION 2 IF ECI4 = 2 OR 3 OR 4 OR 5 OR 6

ONLY DISPLAY RESPONSE OPTION 3 IF ECI4 = 3 OR 4 OR 5 OR 6

ONLY DISPLAY RESPONSE OPTION 4 IF ECI4 = 4 OR 5 OR 6

ONLY DISPLAY RESPONSE OPTION 5 IF ECI4 = 5 OR 6

ONLY DISPLAY RESPONSE OPTION 6 IF ECI4 = 6

Base: if BID287 = 1

BID287LT [S]

[INSERT IMG_287]

How many times have you used the **BIDI® Stick Marigold (**previously known as **BIDI® Stick** **Icy Mango)** in your entire life?

1. 1 time, even just a few puffs

2. 2 to 10 times

3. 11 to 20 times

4. 21 to 50 times

5. 51 to 99 times

6. 100 or more times

SCRIPTER: ONLY DISPLAY RESPONSE OPTION 2 IF ECI4 = 2 OR 3 OR 4 OR 5 OR 6

ONLY DISPLAY RESPONSE OPTION 3 IF ECI4 = 3 OR 4 OR 5 OR 6

ONLY DISPLAY RESPONSE OPTION 4 IF ECI4 = 4 OR 5 OR 6

ONLY DISPLAY RESPONSE OPTION 5 IF ECI4 = 5 OR 6

ONLY DISPLAY RESPONSE OPTION 6 IF ECI4 = 6

Base: if BID288 = 1

BID288LT [S]

[INSERT IMG_288]

How many times have you used the **BIDI® Stick Regal (**previously known as **BIDI® Stick** **Dragon Venom)** in your entire life?

1. 1 time, even just a few puffs

2. 2 to 10 times

3. 11 to 20 times

4. 21 to 50 times

5. 51 to 99 times

6. 100 or more times

SCRIPTER: ONLY DISPLAY RESPONSE OPTION 2 IF ECI4 = 2 OR 3 OR 4 OR 5 OR 6

ONLY DISPLAY RESPONSE OPTION 3 IF ECI4 = 3 OR 4 OR 5 OR 6

ONLY DISPLAY RESPONSE OPTION 4 IF ECI4 = 4 OR 5 OR 6

ONLY DISPLAY RESPONSE OPTION 5 IF ECI4 = 5 OR 6

ONLY DISPLAY RESPONSE OPTION 6 IF ECI4 = 6

Base: if BID289 = 1

BID289LT [S]

[INSERT IMG_289]

How many times have you used the **BIDI® Stick Summer (**previously known as **BIDI® Stick** **Kick Start)** in your entire life?

1. 1 time, even just a few puffs

2. 2 to 10 times

3. 11 to 20 times

4. 21 to 50 times

5. 51 to 99 times

6. 100 or more times

SCRIPTER: ONLY DISPLAY RESPONSE OPTION 2 IF ECI4 = 2 OR 3 OR 4 OR 5 OR 6

ONLY DISPLAY RESPONSE OPTION 3 IF ECI4 = 3 OR 4 OR 5 OR 6

ONLY DISPLAY RESPONSE OPTION 4 IF ECI4 = 4 OR 5 OR 6

ONLY DISPLAY RESPONSE OPTION 5 IF ECI4 = 5 OR 6

ONLY DISPLAY RESPONSE OPTION 6 IF ECI4 = 6

Base: if BID290 = 1

BID290LT [S]

[INSERT IMG_290]

How many times have you used the **BIDI® Stick Solar (**previously known as **BIDI® Stick** **Berry Blast)** in your entire life?

1. 1 time, even just a few puffs

2. 2 to 10 times

3. 11 to 20 times

4. 21 to 50 times

5. 51 to 99 times

6. 100 or more times

SCRIPTER: ONLY DISPLAY RESPONSE OPTION 2 IF ECI4 = 2 OR 3 OR 4 OR 5 OR 6

ONLY DISPLAY RESPONSE OPTION 3 IF ECI4 = 3 OR 4 OR 5 OR 6

ONLY DISPLAY RESPONSE OPTION 4 IF ECI4 = 4 OR 5 OR 6

ONLY DISPLAY RESPONSE OPTION 5 IF ECI4 = 5 OR 6

ONLY DISPLAY RESPONSE OPTION 6 IF ECI4 = 6

Base: if BID291 = 1

BID291LT [S]

[INSERT IMG_291]

How many times have you used the **BIDI® Stick Dawn (**previously known as **BIDI® Stick** **Champion Juice)** in your entire life?

1. 1 time, even just a few puffs

2. 2 to 10 times

3. 11 to 20 times

4. 21 to 50 times

5. 51 to 99 times

6. 100 or more times

SCRIPTER: ONLY DISPLAY RESPONSE OPTION 2 IF ECI4 = 2 OR 3 OR 4 OR 5 OR 6

ONLY DISPLAY RESPONSE OPTION 3 IF ECI4 = 3 OR 4 OR 5 OR 6

ONLY DISPLAY RESPONSE OPTION 4 IF ECI4 = 4 OR 5 OR 6

ONLY DISPLAY RESPONSE OPTION 5 IF ECI4 = 5 OR 6

ONLY DISPLAY RESPONSE OPTION 6 IF ECI4 = 6

**P30DU – BRANDS**

Base: if (ECI5 ≥ 1) AND (ECI8265 = 1)

[M] (Randomize)

Please look carefully at the brand logos below

In the **past 30 days**, have you used any of these brands of e-cigarettes, even once or twice? (**Select all that you have used**)

P30D265. [INSERT IMG_265]

P30D0. I have not used any of these brands of e-cigarettes in the past 30 days.

SCRIPTER: ANCHOR RESPONSE OPTION P30D0 AND MAKE EXCLUSIVE

SCRIPTER: DISPLAY P30D265 IF ECI8265 = 1,

**P30D DEVICE USE – BIDI® Stick**

Base: if (P30D265 = 1) AND (BID281 = 1 OR BID282 = 1 OR BID283 = 1 OR BID284 = 1 OR BID285 = 1 OR BID286 = 1 OR BID287 = 1 OR BID288 = 1 OR BID289 = 1 OR BID290 = 1 OR BID291 = 1)

[M] (Randomize)

Below are pictures of different **BIDI® Stick** e-cigarettes that you said you have used.

In the **past 30 days**, have you used any of these **BIDI® Stick** e-cigarettes, even once or twice? (**Check all that apply**)

P30281. [INSERT IMG_281]

P30282. [INSERT IMG_282]

P30283. [INSERT IMG_283]

P30284. [INSERT IMG_284]

P30285. [INSERT IMG_285]

P30286. [INSERT IMG_286]

P30287. [INSERT IMG_287]

P30288. [INSERT IMG_288]

P30289. [INSERT IMG_289]

P30290. [INSERT IMG_290]

P30291. [INSERT IMG_291]

P30BID0. I have not used any of these e-cigarettes in the past 30 days.

SCRIPTER: ANCHOR RESPONSE OPTION P30BID0 AND MAKE EXCLUSIVE

SCRIPTER:

DISPLAY P30281 IF BID281= 1, DISPLAY P30282 IF BID282= 1, DISPLAY P30283 IF BID283= 1, DISPLAY P30284 IF BID284 = 1, DISPLAY P30285 IF BID285= 1, DISPLAY P30286 IF BID286= 1, DISPLAY P30287 IF BID287 = 1, DISPLAY P30288 IF BID288= 1, DISPLAY P30289 IF BID289 = 1, DISPLAY P30290 IF BID290= 1, DISPLAY P30291 IF BID291= 1

**PATTERNS OF USE – BIDI® Stick ARCTIC**

Base: if P30281 = 1

BID281I [O]

[INSERT IMG_281]

During the **past 30 days,** on how many days did you use the **BIDI® Stick Arctic (**previously known as **BIDI® Stick** **Mint Freeze)**?

1. [SCRIPTER INSERT TEXT BOX] days

Min:1

Max: Response entered in ECI5

CONTROL: NUMERICAL ENTRY ≤ ECI5
SCRIPTER: INSERT NUMERICAL ENTRY BOX TO RESPONSE OPTION 1, NO DECIMALS

Base: if P30281 = 1

BID281D [O]

[INSERT IMG_281]

During the **past 30 days,** about how many **BIDI® Stick Arctic (**previously known as **BIDI® Stick** **Mint Freeze)** e-cigarettes did you use?

If you haven’t used a full **BIDI® Stick Arctic** (Previously known as **BIDI® Stick Mint Freeze**) in the past 30 days, type “0”.

1. [SCRIPTER INSERT TEXT BOX] BIDI® Stick Arctic (Previously known as BIDI® Stick Mint Freeze) e-cigarettes

Min: 0

Max: 60

CONTROL: Max numerical entry 60
SCRIPTER: INSERT NUMERICAL ENTRY BOX TO RESPONSE OPTION 1, NO DECIMALS

Base: if P30281 = 1

BID281Q [S]

[INSERT IMG_281]

Are you seriously thinking about quitting the **BIDI® Stick Arctic (**previously known as **BIDI® Stick** **Mint Freeze)**? (**Please choose the first answer that fits**)

1. Yes, during the next 30 days

2. Yes, during the next 6 months

3. Yes, during the next 12 months

4. Yes, but not during the next 12 months

5. No, I am not thinking about quitting the BIDI® Stick Arctic (Previously known as BIDI® Stick Mint Freeze)

**PATTERNS OF USE – BIDI® Stick CLASSIC**

Base: if P30282 = 1

BID282I [O]

[INSERT IMG_282]

During the **past 30 days,** on how many days did you use the **BIDI® Stick Classic (**previously known as **BIDI® Stick** **Classic Tobacco)**?

1. [SCRIPTER INSERT TEXT BOX] days

Min:1

Max: Response entered in ECI5

CONTROL: NUMERICAL ENTRY ≤ ECI5
SCRIPTER: INSERT NUMERICAL ENTRY BOX TO RESPONSE OPTION 1, NO DECIMALS

Base: if P30282 = 1

BID282D [O]

[INSERT IMG_282]

During the **past 30 days,** about how many **BIDI® Stick Classic (**previously known as **BIDI® Stick** **Classic Tobacco)** e-cigarettes did you use?

If you haven’t used a full **BIDI® Stick Classic** (Previously known as **BIDI® Stick Classic Tobacco**) in the past 30 days, type “0”.

1. [SCRIPTER INSERT TEXT BOX] BIDI® Stick Classic (Previously known as BIDI® Stick Classic Tobacco) e-cigarettes

Min: 0

Max: 60

CONTROL: Max numerical entry 60
SCRIPTER: INSERT NUMERICAL ENTRY BOX TO RESPONSE OPTION 1, NO DECIMALS

Base: if P30282 = 1

BID282Q [S]

[INSERT IMG_282]

Are you seriously thinking about quitting the **BIDI® Stick Classic (**previously known as **BIDI® Stick** **Classic Tobacco)**? (**Please choose the first answer that fits**)

1. Yes, during the next 30 days

2. Yes, during the next 6 months

3. Yes, during the next 12 months

4. Yes, but not during the next 12 months

5. No, I am not thinking about quitting the BIDI® Stick Classic (Previously known as BIDI® Stick Classic Tobacco)

**PATTERNS OF USE – BIDI® Stick ZEST**

Base: if P30283 = 1

BID283I [O]

[INSERT IMG_283]

During the **past 30 days,** on how many days did you use the **BIDI® Stick Zest (**previously known as **BIDI® Stick Jungle Juice)**?

1. [SCRIPTER INSERT TEXT BOX] days

Min:1

Max: Response entered in ECI5

CONTROL: NUMERICAL ENTRY ≤ ECI5
SCRIPTER: INSERT NUMERICAL ENTRY BOX TO RESPONSE OPTION 1, NO DECIMALS

Base: if P30283 = 1

BID283D [O]

[INSERT IMG_283]

During the **past 30 days,** about how many **BIDI® Stick Zest (**previously known as **BIDI® Stick Jungle Juice)** e-cigarettes did you use?

If you haven’t used a full BIDI® Stick Zest (Previously known as BIDI® Stick Jungle Juice) in the past 30 days, type “0”.

1. [SCRIPTER INSERT TEXT BOX] **BIDI® Stick Zest** (Previously known as **BIDI® Stick Jungle Juice**) e-cigarettes

Min: 0

Max: 60

CONTROL: Max numerical entry 60
SCRIPTER: INSERT NUMERICAL ENTRY BOX TO RESPONSE OPTION 1, NO DECIMALS

Base: if P30283 = 1

BID283Q [S]

[INSERT IMG_283]

Are you seriously thinking about quitting the **BIDI® Stick Zest (**previously known as **BIDI® Stick Jungle Juice)**? (**Please choose the first answer that fits**)

1. Yes, during the next 30 days

2. Yes, during the next 6 months

3. Yes, during the next 12 months

4. Yes, but not during the next 12 months

5. No, I am not thinking about quitting the BIDI® Stick Zest (Previously known as BIDI® Stick Jungle Juice)

**PATTERNS OF USE – BIDI® Stick WINTER**

Base: if P30284 = 1

BID284I [O]

[INSERT IMG_284]

During the **past 30 days,** on how many days did you use the **BIDI® Stick Winter (**previously known as **BIDI® Stick** **Lush Ice)**?

1. [SCRIPTER INSERT TEXT BOX] days

Min:1

Max: Response entered in ECI5

CONTROL: NUMERICAL ENTRY ≤ ECI5
SCRIPTER: INSERT NUMERICAL ENTRY BOX TO RESPONSE OPTION 1, NO DECIMALS

Base: if P30284 = 1

BID284D [O]

[INSERT IMG_284]

During the **past 30 days,** about how many **BIDI® Stick Winter (**previously known as **BIDI® Stick** **Lush Ice)** e-cigarettes did you use?

If you haven’t used a full BIDI® Stick Winter (Previously known as BIDI® Stick Lush Ice) in the past 30 days, type “0”.

1. [SCRIPTER INSERT TEXT BOX] **BIDI® Stick Winter** (Previously known as **BIDI® Stick Lush Ice**) e-cigarettes

Min: 0

Max: 60

CONTROL: Max numerical entry 60
SCRIPTER: INSERT NUMERICAL ENTRY BOX TO RESPONSE OPTION 1, NO DECIMALS

Base: if P30284 = 1

BID284Q [S]

[INSERT IMG_284]

Are you seriously thinking about quitting the **BIDI® Stick Winter (**previously known as **BIDI® Stick** **Lush Ice)**? (**Please choose the first answer that fits**)

1. Yes, during the next 30 days

2. Yes, during the next 6 months

3. Yes, during the next 12 months

4. Yes, but not during the next 12 months

5. No, I am not thinking about quitting the BIDI® Stick Winter (Previously known as BIDI® Stick Lush Ice)

**PATTERNS OF USE – BIDI® Stick TROPIC**

Base: if P30285 = 1

BID285I [O]

[INSERT IMG_285]

During the **past 30 days,** on how many days did you use the **BIDI® Stick Tropic (**previously known as **BIDI® Stick** **Blazing Vibe)**?

1. [SCRIPTER INSERT TEXT BOX] days

Min:1

Max: Response entered in ECI5

CONTROL: NUMERICAL ENTRY ≤ ECI5
SCRIPTER: INSERT NUMERICAL ENTRY BOX TO RESPONSE OPTION 1, NO DECIMALS

Base: if P30285 = 1

BID285D [O]

[INSERT IMG_285]

During the **past 30 days,** about how many **BIDI® Stick Tropic (**previously known as **BIDI® Stick** **Blazing Vibe)** e-cigarettes did you use?

If you haven’t used a full BIDI® Stick Tropic (Previously known as BIDI® Stick Blazing Vibe) in the past 30 days, type “0”.

1. [SCRIPTER INSERT TEXT BOX] **BIDI® Stick Tropic** (Previously known as **BIDI® Stick Blazing Vibe**) e-cigarettes

Min: 0

Max: 60

CONTROL: Max numerical entry 60
SCRIPTER: INSERT NUMERICAL ENTRY BOX TO RESPONSE OPTION 1, NO DECIMALS

Base: if P30285 = 1

BID285Q [S]

[INSERT IMG_285]

Are you seriously thinking about quitting the **BIDI® Stick Tropic (**previously known as **BIDI® Stick** **Blazing Vibe)**? (**Please choose the first answer that fits**)

1. Yes, during the next 30 days

2. Yes, during the next 6 months

3. Yes, during the next 12 months

4. Yes, but not during the next 12 months

5. No, I am not thinking about quitting the BIDI® Stick Tropic (Previously known as BIDI® Stick Blazing Vibe)

**PATTERNS OF USE – BIDI® Stick GOLD**

Base: if P30286 = 1

BID286I [O]

[INSERT IMG_286]

During the **past 30 days,** on how many days did you use the **BIDI® Stick Gold (**previously known as **BIDI® Stick** **Fruity Mango)**?

1. [SCRIPTER INSERT TEXT BOX] days

Min:1

Max: Response entered in ECI5

CONTROL: NUMERICAL ENTRY ≤ ECI5
SCRIPTER: INSERT NUMERICAL ENTRY BOX TO RESPONSE OPTION 1, NO DECIMALS

Base: if P30286 = 1

BID286D [O]

[INSERT IMG_286]

During the **past 30 days,** about how many **BIDI® Stick Gold (**previously known as **BIDI® Stick** **Fruity Mango)** e-cigarettes did you use?

If you haven’t used a full **BIDI® Stick Gold** (Previously known as **BIDI® Stick Fruity Mango**) in the past 30 days, type “0”.

1. [SCRIPTER INSERT TEXT BOX] BIDI® Stick Gold (Previously known as BIDI® Stick Fruity Mango) e-cigarettes

Min: 0

Max: 60

CONTROL: Max numerical entry 60
SCRIPTER: INSERT NUMERICAL ENTRY BOX TO RESPONSE OPTION 1, NO DECIMALS

Base: if P30286 = 1

BID286Q [S]

[INSERT IMG_286]

Are you seriously thinking about quitting the **BIDI® Stick Gold (**previously known as **BIDI® Stick** **Fruity Mango)**? (**Please choose the first answer that fits**)

1. Yes, during the next 30 days

2. Yes, during the next 6 months

3. Yes, during the next 12 months

4. Yes, but not during the next 12 months

5. No, I am not thinking about quitting the BIDI® Stick Gold (Previously known as BIDI® Stick Fruity Mango)

**PATTERNS OF USE – BIDI® Stick MARIGOLD**

Base: if P30287 = 1

BID287I [O]

[INSERT IMG_287]

During the **past 30 days,** on how many days did you use the **BIDI® Stick Marigold (**previously known as **BIDI® Stick** **Icy Mango)**?

1. [SCRIPTER INSERT TEXT BOX] days

Min:1

Max: Response entered in ECI5

CONTROL: NUMERICAL ENTRY ≤ ECI5
SCRIPTER: INSERT NUMERICAL ENTRY BOX TO RESPONSE OPTION 1, NO DECIMALS

Base: if P30287 = 1

BID287D [O]

[INSERT IMG_287]

During the **past 30 days,** about how many **BIDI® Stick Marigold (**previously known as **BIDI® Stick** **Icy Mango)** e-cigarettes did you use?

If you haven’t used a full **BIDI® Stick Marigold** (Previously known as **BIDI® Stick Icy Mango**) in the past 30 days, type “0”.

1. [SCRIPTER INSERT TEXT BOX] BIDI® Stick Marigold (Previously known as BIDI® Stick Icy Mango) e-cigarettes

Min: 0

Max: 60

CONTROL: Max numerical entry 60
SCRIPTER: INSERT NUMERICAL ENTRY BOX TO RESPONSE OPTION 1, NO DECIMALS

Base: if P30287 = 1

BID287Q [S]

[INSERT IMG_287]

Are you seriously thinking about quitting the **BIDI® Stick Marigold (**previously known as **BIDI® Stick** **Icy Mango)**? (**Please choose the first answer that fits**)

1. Yes, during the next 30 days

2. Yes, during the next 6 months

3. Yes, during the next 12 months

4. Yes, but not during the next 12 months

5. No, I am not thinking about quitting the BIDI® Stick Marigold (Previously known as BIDI® Stick Icy Mango)

**PATTERNS OF USE – BIDI® Stick REGAL**

Base: if P30288 = 1

BID288I [O]

[INSERT IMG_288]

During the **past 30 days,** on how many days did you use the **BIDI® Stick Regal (**previously known as **BIDI® Stick** **Dragon Venom)**?

1. [SCRIPTER INSERT TEXT BOX] days

Min:1

Max: Response entered in ECI5

CONTROL: NUMERICAL ENTRY ≤ ECI5
SCRIPTER: INSERT NUMERICAL ENTRY BOX TO RESPONSE OPTION 1, NO DECIMALS

Base: if P30288 = 1

BID288D [O]

[INSERT IMG_288]

During the **past 30 days,** about how many **BIDI® Stick Regal (**previously known as **BIDI® Stick** **Dragon Venom)** e-cigarettes did you use?

If you haven’t used a full **BIDI® Stick Regal** (Previously known as **BIDI® Stick Dragon Venom**) in the past 30 days, type “0”.

1. [SCRIPTER INSERT TEXT BOX] BIDI® Stick Regal (Previously known as BIDI® Stick Dragon Venom) e-cigarettes

Min: 0

Max: 60

CONTROL: Max numerical entry 60
SCRIPTER: INSERT NUMERICAL ENTRY BOX TO RESPONSE OPTION 1, NO DECIMALS

Base: if P30288 = 1

BID288Q [S]

[INSERT IMG_288]

Are you seriously thinking about quitting the **BIDI® Stick Regal (**previously known as **BIDI® Stick** **Dragon Venom)**? (**Please choose the first answer that fits**)

1. Yes, during the next 30 days

2. Yes, during the next 6 months

3. Yes, during the next 12 months

4. Yes, but not during the next 12 months

5. No, I am not thinking about quitting the BIDI® Stick Regal (Previously known as BIDI® Stick Dragon Venom)

**PATTERNS OF USE – BIDI® Stick SUMMER**

Base: if P30289 = 1

BID289I [O]

[INSERT IMG_289]

During the **past 30 days,** on how many days did you use the **BIDI® Stick Summer (**previously known as **BIDI® Stick** **Kick Start)**?

1. [SCRIPTER INSERT TEXT BOX] days

Min:1

Max: Response entered in ECI5

CONTROL: NUMERICAL ENTRY ≤ ECI5
SCRIPTER: INSERT NUMERICAL ENTRY BOX TO RESPONSE OPTION 1, NO DECIMALS

Base: if P30289 = 1

BID289D [O]

[INSERT IMG_289]

During the **past 30 days,** about how many **BIDI® Stick Summer (**previously known as **BIDI® Stick** **Kick Start)** e-cigarettes did you use?

If you haven’t used a full **BIDI® Stick Summer** (Previously known as **BIDI® Stick Kick Start**) in the past 30 days, type “0”.

1. [SCRIPTER INSERT TEXT BOX] BIDI® Stick Summer (Previously known as BIDI® Stick Kick Start) e-cigarettes

Min: 0

Max: 60

CONTROL: Max numerical entry 60
SCRIPTER: INSERT NUMERICAL ENTRY BOX TO RESPONSE OPTION 1, NO DECIMALS

Base: if P30289 = 1

BID289Q [S]

[INSERT IMG_289]

Are you seriously thinking about quitting the **BIDI® Stick Summer (**previously known as **BIDI® Stick** **Kick Start)**? (**Please choose the first answer that fits**)

1. Yes, during the next 30 days

2. Yes, during the next 6 months

3. Yes, during the next 12 months

4. Yes, but not during the next 12 months

5. No, I am not thinking about quitting the BIDI® Stick Summer (Previously known as BIDI® Stick Kick Start)

**PATTERNS OF USE – BIDI® Stick SOLAR**

Base: if P30290 = 1

BID290I [O]

[INSERT IMG_290]

During the **past 30 days,** on how many days did you use the **BIDI® Stick Solar (**previously known as **BIDI® Stick** **Berry Blast)**?

1. [SCRIPTER INSERT TEXT BOX] days

Min:1

Max: Response entered in ECI5

CONTROL: NUMERICAL ENTRY ≤ ECI5
SCRIPTER: INSERT NUMERICAL ENTRY BOX TO RESPONSE OPTION 1, NO DECIMALS

Base: if P30290 = 1

BID290D [O]

[INSERT IMG_290]

During the **past 30 days,** about how many **BIDI® Stick Solar (**previously known as **BIDI® Stick** **Berry Blast)** e-cigarettes did you use?

If you haven’t used a full **BIDI® Stick Solar** (Previously known as **BIDI® Stick Berry Blast**) in the past 30 days, type “0”.

1. [SCRIPTER INSERT TEXT BOX] BIDI® Stick Solar (Previously known as BIDI® Stick Berry Blast) e-cigarettes

Min: 0

Max: 60

CONTROL: Max numerical entry 60
SCRIPTER: INSERT NUMERICAL ENTRY BOX TO RESPONSE OPTION 1, NO DECIMALS

Base: if P30290 = 1

BID290Q [S]

[INSERT IMG_290]

Are you seriously thinking about quitting the **BIDI® Stick Solar (**previously known as **BIDI® Stick** **Berry Blast)**? (**Please choose the first answer that fits**)

1. Yes, during the next 30 days

2. Yes, during the next 6 months

3. Yes, during the next 12 months

4. Yes, but not during the next 12 months

5. No, I am not thinking about quitting the BIDI® Stick Solar (Previously known as BIDI® Stick Berry Blast)

**PATTERNS OF USE – BIDI® Stick DAWN**

Base: if P30291 = 1

BID291I [O]

[INSERT IMG_291]

During the **past 30 days,** on how many days did you use the **BIDI® Stick Dawn (**previously known as **BIDI® Stick** **Champion Juice)**?

1. [SCRIPTER INSERT TEXT BOX] days

Min:1

Max: Response entered in ECI5

CONTROL: NUMERICAL ENTRY ≤ ECI5
SCRIPTER: INSERT NUMERICAL ENTRY BOX TO RESPONSE OPTION 1, NO DECIMALS

Base: if P30291 = 1

BID291D [O]

[INSERT IMG_291]

During the **past 30 days,** about how many **BIDI® Stick Dawn (**previously known as **BIDI® Stick** **Champion Juice)** e-cigarettes did you use?

If you haven’t used a full **BIDI® Stick Dawn** (Previously known as **BIDI® Stick Champion Juice**) in the past 30 days, type “0”.

1. [SCRIPTER INSERT TEXT BOX] BIDI® Stick Dawn (Previously known as BIDI® Stick Champion Juice) e-cigarettes

Min: 0

Max: 60

CONTROL: Max numerical entry 60
SCRIPTER: INSERT NUMERICAL ENTRY BOX TO RESPONSE OPTION 1, NO DECIMALS

Base: if P30291 = 1

BID291Q [S]

[INSERT IMG_291]

Are you seriously thinking about quitting the **BIDI® Stick Dawn (**previously known as **BIDI® Stick** **Champion Juice)**? (**Please choose the first answer that fits**)

1. Yes, during the next 30 days

2. Yes, during the next 6 months

3. Yes, during the next 12 months

4. Yes, but not during the next 12 months

5. No, I am not thinking about quitting the BIDI® Stick Dawn (Previously known as BIDI® Stick Champion Juice)

Base: All respondents

END_INT

Thank you for taking part in our survey we appreciate your time.
